# Edaphic properties as pieces of evidence of tailings deposit on soils

**DOI:** 10.1007/s10653-023-01657-x

**Published:** 2023-06-25

**Authors:** Diana Zúñiga-Vázquez, María Aurora Armienta, Olivia Cruz, Alejandra Aguayo, Isabel Pérez-Martínez, José Iván Morales-Arredondo

**Affiliations:** 1 Universidad Nacional Autónoma de México, Instituto de Geofísica, UNAM, 04510 Mexico City, Mexico; 2 CONACyT-Instituto de Geofísica, UNAM, 04510 Mexico City, Mexico

**Keywords:** Soils, Mine tailings, Edaphic parameters, Zimapán, Potentially toxic elements

## Abstract

Mine tailings are one of the primary contaminant sources of heavy metals and metalloids in the soil. Besides increasing the concentration of potentially toxic elements (PTEs), tailings may modify the edaphic conditions and decrease the buffer capacity of impacted soils. The influence of tailings may reach distances far from the impoundments depending on the transport path and the specific transport mean: air, rain (runoff and infiltration), or acid mine drainage. In this study, soil samples from various horizons were collected in trial pits along a transect, at different distances from sulfide tailings. Soil analysis included texture, organic matter, alkalinity, porous space, carbonates, pH, electrical conductivity, real density, apparent density, total sulfur, main mineralogy, and total concentrations of As, Cd, Pb, Fe, and Zn. Graphical and statistical interpretation of the results showed that real density and porous space are the leading indicators of the tailings dispersion and accumulation and that pH is not a significant parameter (all values were above the neutrality) due to the limestone abundance in the area. However, Zn and Cd concentrations had an inverse relation with pH. Differences in the concentrations of PTEs between the superficial and deep layers that increased toward the tailings were also observed. Gypsum was only present in the closest samples to the tailings and may also be an indicator of tailings’ influence on soils. This study allowed us to identify general edaphic parameters as a first and quick means to determine the tailings contamination of soils.

**Supplementary Information:**

The online version contains supplementary material available at 10.1007/s10653-023-01657-x.

## Introduction

Soil is a dynamic body that changes its physical, chemical, and biological properties according to the factors that formed it, giving it specific characteristics. It develops from a mixture of minerals and organic matter under the influence of climate and environment (Kabata-Pendias, 2011). However, the soil is vulnerable to human activities, either by excessive addition of substances (such as fertilizers) or by being exposed to different sources of pollutants, among which mining activity stands out (Bai et al., 2021; Mazurek et al., 2017; Mourinha et al., 2022; Wuana & Okieimen, 2011).

The mining industry generates wastes called tailings, which contain high concentrations of potentially toxic elements (heavy metals and metalloids). These wastes can be dispersed by physical means (wind and rain) and increase their mobility (Olobatoke & Mathuthu, 2016), causing their deposition in the surrounding soils and consequent contamination of these; in such a way that, as the wastes interact with the different phases of the soil, they give rise to secondary mineralogy derived from processes such as precipitation (Nordstrom & Alpers, 1999). The mineral composition of these residues and the soil is a relevant factor for the mobility of potentially toxic elements (Li & Thornton, 2001; Rivera et al., 2015). Secondary minerals formed in soils, together with organic matter, are the most active part of the soil, where the presence of a contaminant and its accumulation will negatively affect its functions (Bauer et al., 2019; Mazurek et al, 2017). The transport by air is influenced by the direction and velocity of winds, orography, vegetation, other natural and manufactured barriers, and the climate, which also affects the transport by water. The precipitation regime, soil permeability, presence of water bodies, and orography influence the tailings transport by runoff. The generation of acid mine drainage from sulfide oxidation within tailings and in soils impacted by sulfides from these wastes represents another potentially toxic elements (PTEs) transport mean.

Heavy metal contamination is known to modify soil quality by affecting its properties, physical, chemical, and biological (Bauer et al., 2019; Ciarkowska & Gambus, 2020) and this has an impact on its buffering capacity (Baran et al., 2017; Bauer et al., 2019; Vega et al, 2004). Heavy metals are non-biodegradable, so they can accumulate and present high contents in surface soil layers (Acosta et al., 2015; Bai et al., 2021; Chu, 2018; Hu et al., 2021; Mazurek et al, 2017; Mourinha et al., 2022). On the other hand, the physical properties of soil can alter the chemical forms of PTEs species (Hu et al., 2021) and modify their bioavailability, increasing the potential toxicological risk of soils contaminated by mining waste. In addition, the tailings particulates and solutions may affect the soils, not only by increasing the concentrations of PTEs but also by modifying the edaphic properties such as texture disequilibrium, absence or low edaphic structure, decrease or disequilibrium of nutrients, root difficulties, disruption of biogeochemical cycles, lower exchange capacity, and retention or water infiltration (Puga et al., 2006).

**Fig. 1:**
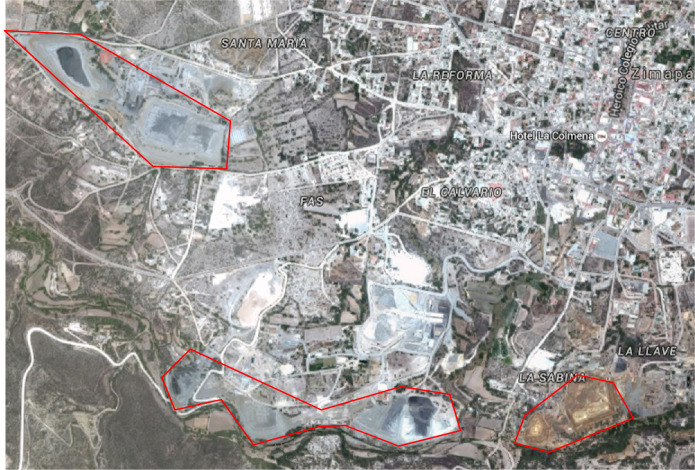
Digital Globe 2016. Google Earth, showing the tailings impoundments (surrounded with a red line) at Zimapán, Mexico

The alteration of physical properties (texture, pore space, real and apparent density, etc.) and organic matter content in soils due to the presence of PTEs may indicate dispersion, deposition, and contamination of mining wastes. However, most of the studies focus on changes in soil chemical and biological properties as evidence of PTEs contamination. Therefore, this work is aimed to analyze the different physical, chemical, and mineralogical variables of soils possibly impacted by tailings at Zimapán, central Mexico, to identify the best indicators of PTEs contamination of soils, derived from the dispersion and deposition of mining wastes. At Zimapán, tailings from the flotation process have accumulated along the town skirts representing an environmental risk to the population (Fig. 1). Although various studies have reported the presence and distribution of PTEs in soils surrounding the tailings deposits (Armienta et al., 2016; Ongley et al., 2007), their impact on the edaphic properties has not been assessed, nor has their modification with distance owing to the particulates transport by air or runoff. This study determined the physicochemical characteristics of possibly impacted soils along a transect from a site next to two tailings impoundments up to 325 m far, crossing a stream to the top of a small hill.

## Study site

Zimapán Hidalgo municipality is a historical mining site located in central Mexico between 20°34′ and 20°50′ North latitude, and 99°11′ and 99°33′ West longitude, between 900 and 2900 m asl. Temperature varies from 12 to 20° C, and precipitation ranges from 400 to 1100 mm/year, mainly as storms (INEGI, 2010). The geology comprises Las Trancas Formation Upper Jurassic calcareous shales, which is overlaid by the Cretaceous limestones Tamaulipas, Abra, and Soyatal Formations. In addition, Tertiary Age continental and volcanic rocks El Morro Fanglomerate and Las Espinas Formation are also present, mainly in the central and east of the area. Late Tertiary and Quaternary alluvial fans cover the lower zones (García & Querol, 1991; Simons & Mapes-Vazquez, 1956). In the conglomerate zone, Leptosols are the more abundant soils (43%), Regosols (25%) are present to the north, while Phaeozems (17.53%) followed by Luvisols (11%) and Calcisols (2%) occur in the valley area (INEGI, 2010). The urban zone covers alluvial quaternary soils that previously corresponded to Regosol, Rendzina, Calcisol, Leptosol, and Phaeozem (INEGI, 1972, 2010).

Primary mineralogy of the orebody includes galena (PbS), sphalerite (ZnS), pyrite (FeS_2_), chalcopyrite (CuFeS_2_), arsenopyrite (AsFeS), pyrrotite (Fe_1-x_S), bornite (Cu_5_FeS_4_), stibinite (Sb_2_S_3_), boulangerite (Pb_5_Sb_4_S_11_), wollastonite (CaSiO_3_), andradite [Ca_3_Fe_2_(SiO_4_)_3_], diopside [CaMg(SiO_3_)_2_], calcite (CaCO_3_), Pb and Ag sulfosalts, jamesonite (Pb_4_FeSb_6_S_14_), and freibergite [(Ag,Cu,Fe)_12_(Sb,As)_4_S_13_] (Moreno Tovar et al., 2009; Villaseñor et al., 1987).

At Zimapán town, high concentrations of arsenic have been reported in the groundwater of deep wells due to natural processes resulting from water interaction with As-rich minerals (Armienta et al., 1997, 2001; Sracek et al., 2010). In addition, tailings accumulated in the west and southwest of the urban zone have polluted shallow wells with As (Armienta et al., 1997). Tailings have also impacted the surrounding soils with PTEs at Zimapán (Armienta et al., 2016; Ongley et al., 2007). Bioaccumulation of PTEs, besides toxic effects (delayed growth and physiological immaturity of cobs), has been observed on maize plants grown in soils near tailings impoundments (Armienta et al., 2020). Arsenic bioaccumulation was also reported in the stages of development of malt barley grown in Zimapán soils irrigated with As-rich water from the local water tank (Prieto García et al., 2010).

In the present study, soil samples were collected in a catena starting next to the basement of two tailings impoundments produced from the Zn–Pb–Ag–(Cu) skarn deposit processing and accumulated for more than 50 years (Moreno Tovar et al., 2009). Primary minerals identified in tailings include arsenopyrite, galena, calcite, sphalerite, sylvanite, beudantite, and quartz (Mendez and Armienta, 2003; Zúñiga-Vázquez et al., 2019). Secondary minerals formed in tailings include jarosite [KFe_3_(SO_4_)_2_(OH)_6_], plumbojarosite [(Pb_0.5_Fe_3_(SO_4_)_2_(OH_6_)], gypsum (SO_4_Ca^.^2H_2_O), anglesite (PbSO_4_), makovikyte (Ag_1.5_Bi_5.5_S_9_) (Zúñiga Vázquez, 2014), goethite [αFeO(OH)], and cerusite (PbCO_3_) (Moreno Tovar et al., 2009).

## Methods

### Soil sampling

**Fig. 2:**
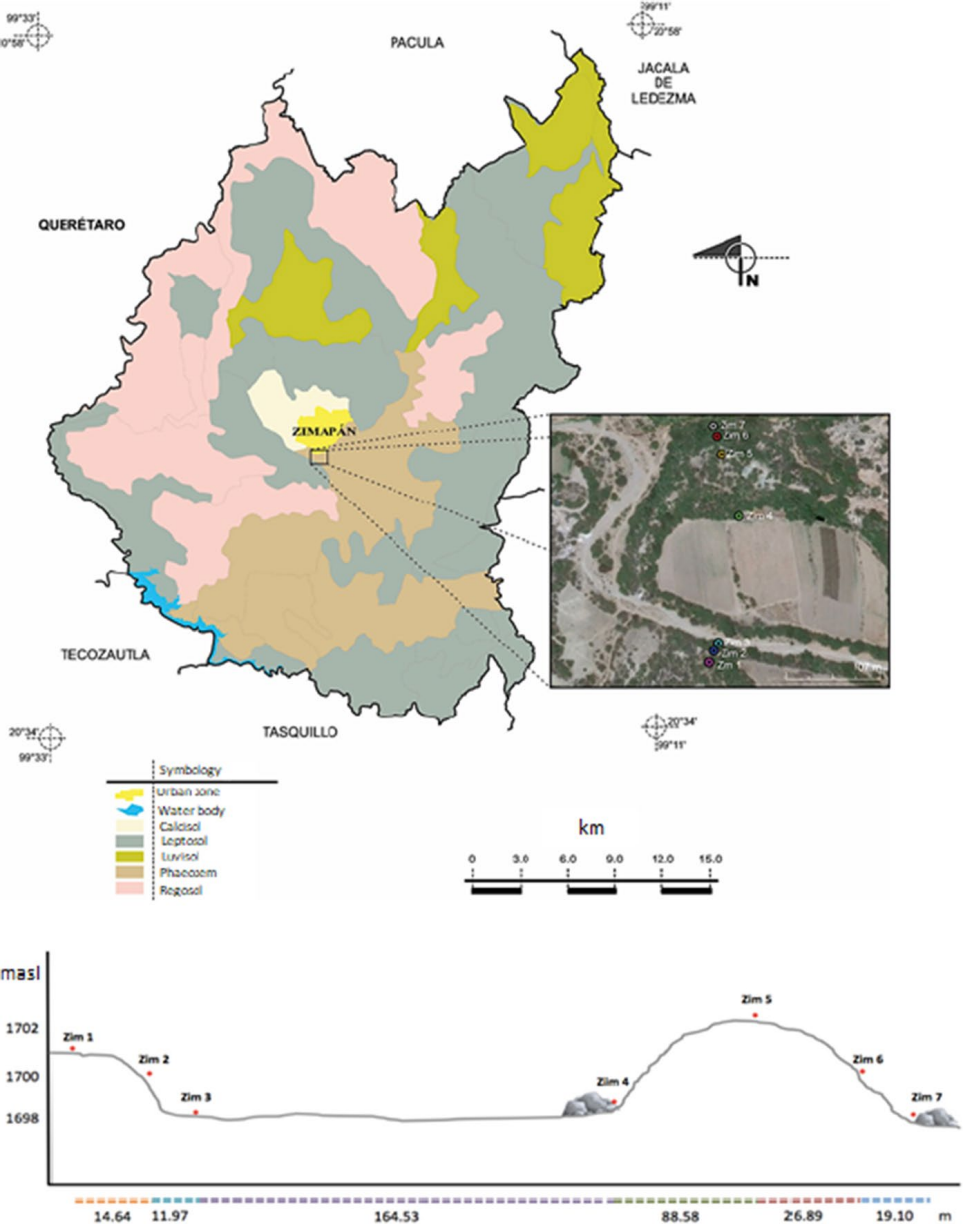
Map of soils at Zimapán Municipality (INEGI, 2010) indicating the sampled points. Profile of the sampled locations (below)

Soil sampling sites were located along a straight line starting from two large tailings impoundments to the west (Fig. 2). Point Zim 7 corresponds to the site closest to the impoundments and Zim 1 to the farthest. At each point, the location was determined with a GPS II Plus Garmin Personal Navigator, and the soil profiles were identified, described, and sampled (simple samples from each soil horizon).

### Physicochemical and mineralogical determinations

The preparation and physicochemical characterization of the soil followed Mexican regulation NOM-021-SEMARNAT-2000 (2002). The color was determined by comparing it with the Munsell color chart. Granulometry was determined with the pipette method obtaining the sand, silt, and clay percentages. Organic matter was measured following the Walkley (1947) chromic acid wet oxidation method modified by Jackson (1958). Alkalinity and carbonates in the soil solution were determined by titration (APHA, 2005) in the solution obtained by adding 20 mL deionized water to 1 g soil, agitating for 18 h, and filtrating through 0.45 µm. Calcium carbonate was also estimated in the field by observing the effervescence of the soil treated with a 10% HCl solution. Electrical conductivity and pH were measured following the USEPA 9045D method in a 1:1 water/soil mixture and measured with a Conductronic PC18. Real density (relation between soil weight and particle volume without considering void space) was measured following the Mexican standard procedure NOM-147-SEMARNAT/SSA1-2004 (2007). Apparent density, weight per unit volume considering the void space, was determined with the cylinder method (Casanova Olivo, 2005). Porous space was calculated from the apparent and particle density (Arias Jiménez, 2007). To analyze the concentrations of metals by atomic absorption spectrometry, 0.5 g soil was slowly added with 5 mL H_2_O_2_, followed by 10 mL aqua regia (1:3 HNO_3_:HCl) and digested in a microwave oven CEM MARSXpress, then filtrated through 0.45 µm and taken to a 50 mL volume. Analytical determinations were performed with a Perkin Elmer AAnalyst 100 Atomic Absorption spectrophotometer, Pb, Cd, Fe, and Zn concentrations by flame, and As by hydride generation with a FIAS 100. Analytical quality included measuring duplicates, blanks, and checking the calibration every 10 samples. Standards were prepared with High Purity standards (NIST traceable) solutions. Accuracy was determined by analyzing NIST Montana II soil 2711a. Average recoveries were: As (79.6%), Cd (67.1%), Pb (75.4%), Fe (67%), and Zn (80.4%). Total sulfur was determined by infrared absorption with a LECO S-44. The standard Leco “Sulfur in coal reference material” Part No. 502-435, sulfur 1.09% (±) 0.05% was used for calibration. Mineralogy was determined with a diffractometer Siemens D5000 using CuKα *λ* = 1.54 Å radiation, 2θ scan range 4–70°. The software TRACES was used for the interpretation of diffractograms.

## Results and discussion

**Fig. 3:**
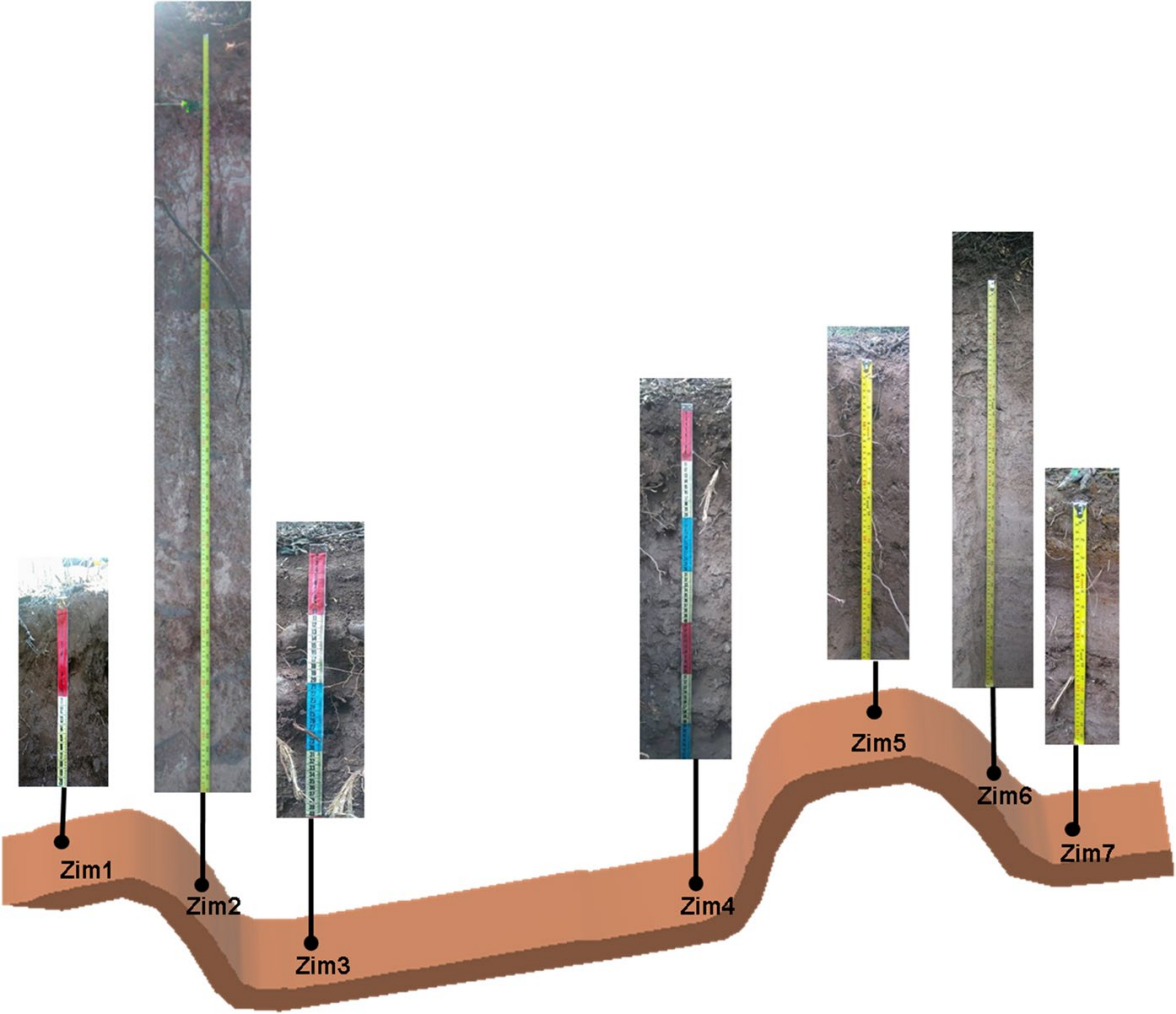
Schematic graph of the catena and pictures of the soil profiles. Sampled depths and horizons are reported in Table 1

The depth of the profiles ranged from 20 to 185 cm without a trend. A schematic graph of the location and pictures of the profiles is shown in Fig. 3. Sampled depths, horizons, and PTE concentrations for each profile are reported in Table 1. Results from the farthest (Zim 1) and closest (Zim 7) to tailings soil profiles are detailed next as an example of the information obtained from each site.

**Table 1:** Soil horizons and concentrations (mg/kg) of As, Pb, Zn, Fe, and Cd in each profile and depth

Depth (cm)	Horizons	As (mg/kg)	Pb(mg/kg)	Zn (mg/kg)	Fe (mg/kg)	Cd (mg/kg)
*Zim 1*						
0–3	Ap	531.5	1065	637.5	22,993	7
3–11	A	205.9	724.5	325.0	16,243	5.8
11–20	AB	75.5	184.8	98.8	23,118	1.5
*Zim 2*						
0–4	Ah	115.8	340.0	275.0	21,368	3.5
4–10	A	31.2	103.2	75.0	20,056	1.5
10–18	AB	24.6	11.2	49.4	21,743	0.8
18–28	B_1_	22.2	7.5	60.6	20,868	0.5
28–39	B_2_	28.3	2.5	45.6	15,868	1.0
39–66	BC_1_	4.1	9.0	52.5	18,431	1.0
66–127	BC_2_	2.9	7.2	56.3	20,243	1.0
127–167	BCk	5.1	24.0	77.5	26,306	1.0
167–185	C	2.9	3.2	53.8	14,181	1.5
*Zim 3*						
0–4	A	35.9	36.0	77.5	22,306	1.5
4–7	B	41.8	38.5	80.6	23,431	1.5
7–40	BC	7.5	15.2	45.6	16,556	1.5
*Zim 4*						
0–6	Ap	518.9	670.0	512.5	25,806	7.0
6–16	A	357.2	1017.5	500.0	23,243	9.8
16–24	B_1_	206.8	1148.9	362.5	22,806	11.1
24–34	B_2_	119.1	555.0	15.6	20,493	1.0
34–51	BC	115.6	562.2	125.0	20,743	3.0
51–68	C	83.6	357.5	112.5	21,868	1.8
*Zim 5*						
0–5	Ap	128.4	156.0	300.0	13,993	2.0
5–20	BA	168	283.0	135.0	15,243	3.2
20–31	B_C_	215.9	122.2	109.4	17,868	1.5
31–45	BC	534.8	238.0	115.0	17,243	3.0
45–56	CB	117.4	15.8	58.8	16,806	0.5
*Zim 6*						
0–5	Ah	1484.5	1082.5	1550.0	42,493	14.5
5–10	BA	153.0	15.2	68.8	21,368	1.0
10–26	B_1_	193.2	5.0	52.5	16,556	1.0
26–42	B_2_	229.4	4.8	65.0	16,868	1.2
42–55	BC	177.9	5.8	56.9	13,118	1.0
55–87	C	23.7	7.2	83.8	28,993	< 0.5
*Zim 7*						
0–3	Ag	1092.5	1022.5	675.0	41,243	5.5
3–9	Cg	4368.0	1490.6	2100.0	62,493	18.5
9–14	CBg	426.4	710.0	600.0	38,493	4.0
14–24	BCg_1_	1728.8	825.0	800.0	38,743	7.5
24–36	BCg_2_	905.8	847.5	850.0	30,118	10.5
36–44	CB	97.2	111.8	76.2	21,118	1.0

### Zim 1

**Fig. 4:**
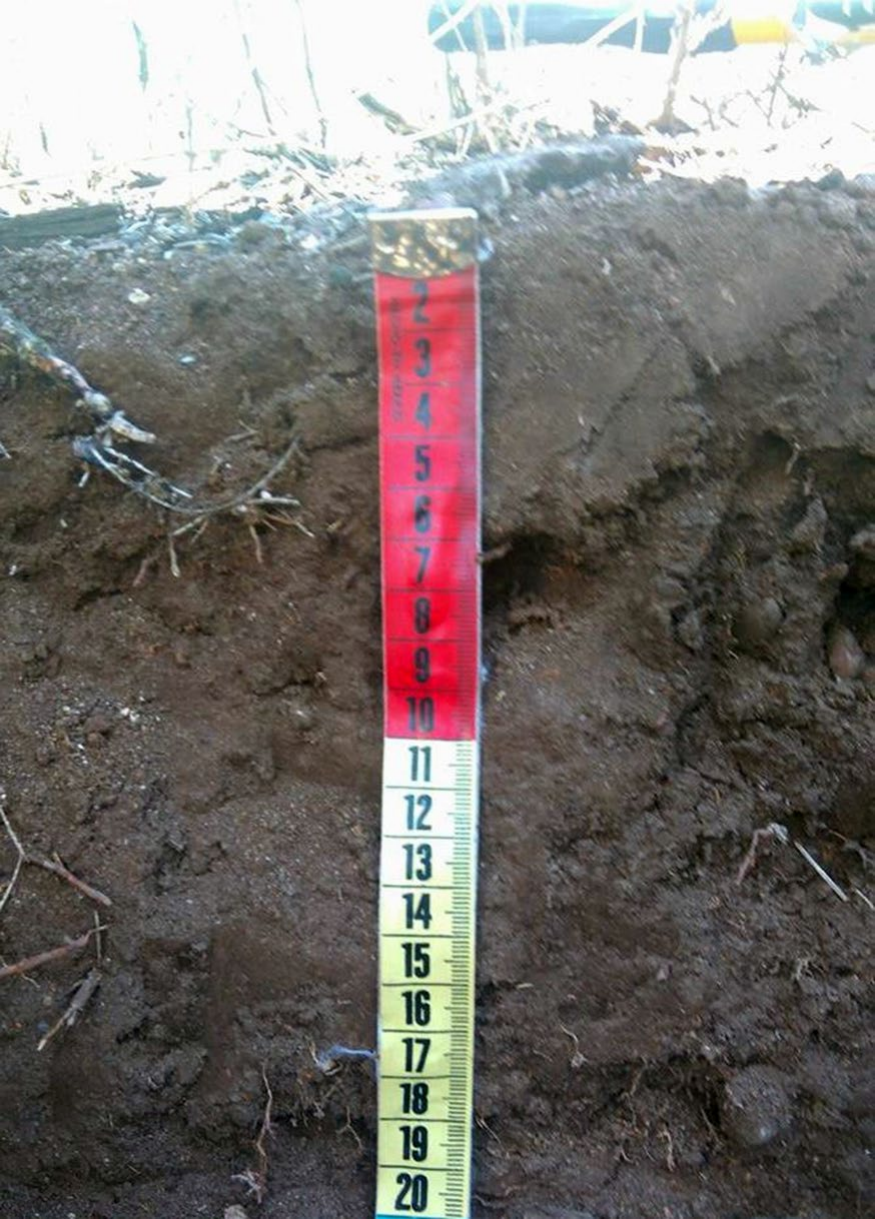
Zim 1 profile

This profile was obtained from the farthest site (20°43′21′′ N 99°23′11′′ W), approximately 326.7 m from the tailings impoundments (Figs. 2, 3) crossing a first hill, a stream, at the top of a second hill. The profile was dug in a site with a slight slope (2%) facing the north. This soil profile was located in an area that was used first for agriculture, then for grazing, and currently, the wild grass is constantly burnt. The profile was shallow (20 cm) with good drainage, gray-brown color, excessively stony (60–80%), and without carbonate evidence. The structure was simple grain at the surface and granular at depth (picture shown in Fig. 4). The soil profile showed low stability of aggregates, micro to very fine interstitial pores, low root density, and diffuse limits. Three horizons (Ap 0–3 cm, A 3–11 cm, Ab 11–20 cm) were identified with real 2.98, 2.15, 2.43 g/cm^3^ and apparent 1.3, 1.14, 1.1 g/cm^3^ densities, respectively. The porous space varied as 56% > 47%  < 54%, clay contents as 26% > 22% < 31%, and conductivity as 0.2 > 0.1 < 0.2 mS/cm from 1 to 3 horizons. Organic matter content varied from 2.66 to 1.43 and 1.98% from horizons 1–3. Alkalinity had the same values in horizons 1 and 2 with 100 mg/kg CaCO_3_ and increased in horizon 3 with 180 mg/kg CaCO_3_, pH varied as 6.9, 6.6, and 7.9. The basic pH measured in horizon 3 is related to a high CaCO_3_ content in this layer (51,260 mg/kg).

**Fig. 5:**
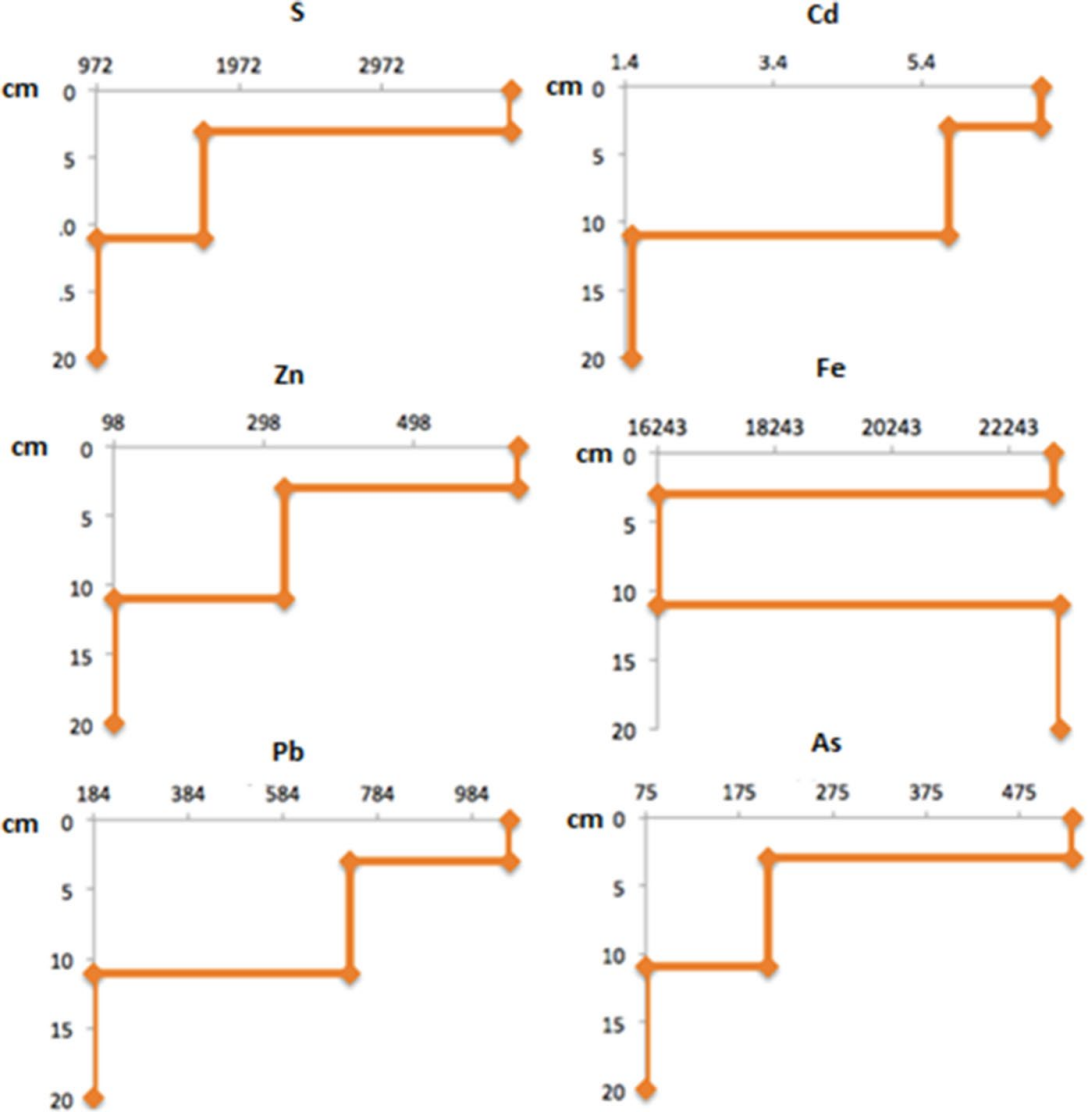
Concentrations (mg/kg) of S, Cd, Zn, Fe, Pb, and As with depth (cm) in Zim 1 soil profile

All the analyzed elements (S, Cd, Pb, Fe As, and Zn) except Fe showed the same behavior decreasing at depth (Table 1). The concentration of Pb was above that established in Mexican standards (NOM-147) for agricultural soils (450 mg/kg) and close to the industrial limit (700 mg/kg) in horizon 2. Arsenic concentrations surpass the industrial (260 mg/kg) and agricultural (22 mg/kg) limits in the three horizons, while Cd was below the standards (37 mg/kg and 450 mg/kg for agricultural and industrial use, respectively) in the three horizons (Fig. 5). The Zn concentration limit is not included in NOM-147.

### Zim 7

**Fig. 6:**
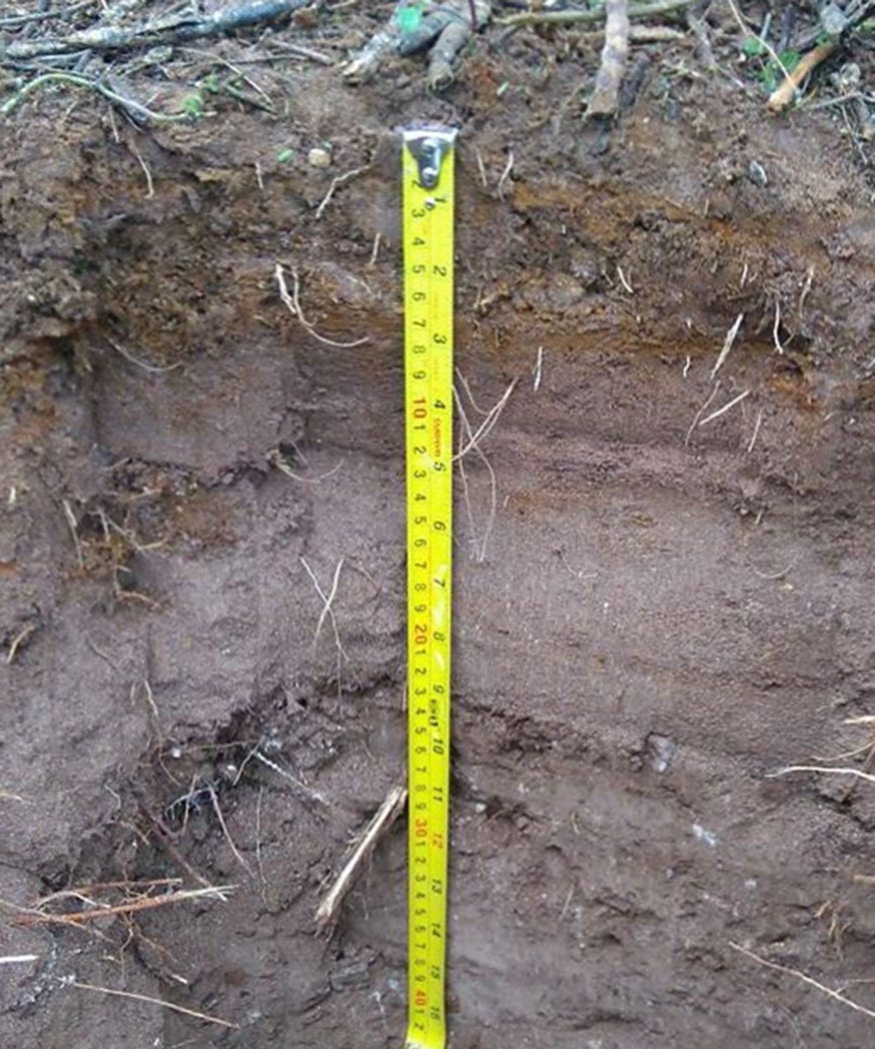
Zim 7 profile

This profile was obtained from the closest site (20°43′31.67′′ N 99°23′9.29′′ W) to the tailings deposits, next to the base of one of the impoundments (Picture shown in Fig. 3). The site is located at the foot of the hill with 35° slope, exposed to the west. The studied profile was shallow (44 cm) and highly stony (40%) in the 4th horizon with a massive structure at the surface with angular blocks at depth. Few interstitial pores range from fine to very fine. Low to very low root density also was observed in this profile. The soil had a reaction to phenolphthalein and with HCl. A low–average aggregates stability was observed along the profile. Six horizons were identified (Ag 0–3 cm, Cg 3–9 cm, CBg 9–14 cm, BCg_1_ 14–24 cm, BCg_2_ 24–36 cm, CB 36–44 cm). A light and continuous tailings layer was observed in Cg horizon (Fig. 6). This profile showed the most heterogeneous physicochemical characteristics of all the studied profiles. Apparent density varied as 1.12, 0.83, 0.9, 1.09, 1.01, and 1.08 g/cm^3^ from the first to the sixth horizon, respectively. Real density values were: 3.13, 3.41, 2.47, 2.19, and 2.54 g/cm^3^, and porous space 64.1, 75.6, 63.5, 50.1, 60.1, and 52.3% from horizons 1 to 6, respectively. The clay content was 19.1, 56.7, and 32.3% in the first three horizons. Carbonates determined in the soil solution varied as 40, 40, 60, 80, 60, and 60 mg/L CaCO_3_, and bicarbonates as 48.8, 48.8, 73.2, 97.6, 73.2, and 73.2 mg/L HCO_3_^−^ from horizons 1 to 6, respectively. The highest conductivity was determined in the superficial layers with 2.7 and 3 mS/cm in horizons 1 and 2, decreasing to 1.7, 0.7, 2.1, and 0.1 mS/cm in the deepest horizons. Carbonates varied as 63,740, 61,940, 66,200, 66,380, 63,020, and 62,960 mg/kg from the 1st to the 6th horizon. The pH ranged from near neutral to slightly alkaline; 7.3, 6.9, 7.3, 7.6, 7.4, and 7.7 from the horizon 1–6.

**Fig. 7:**
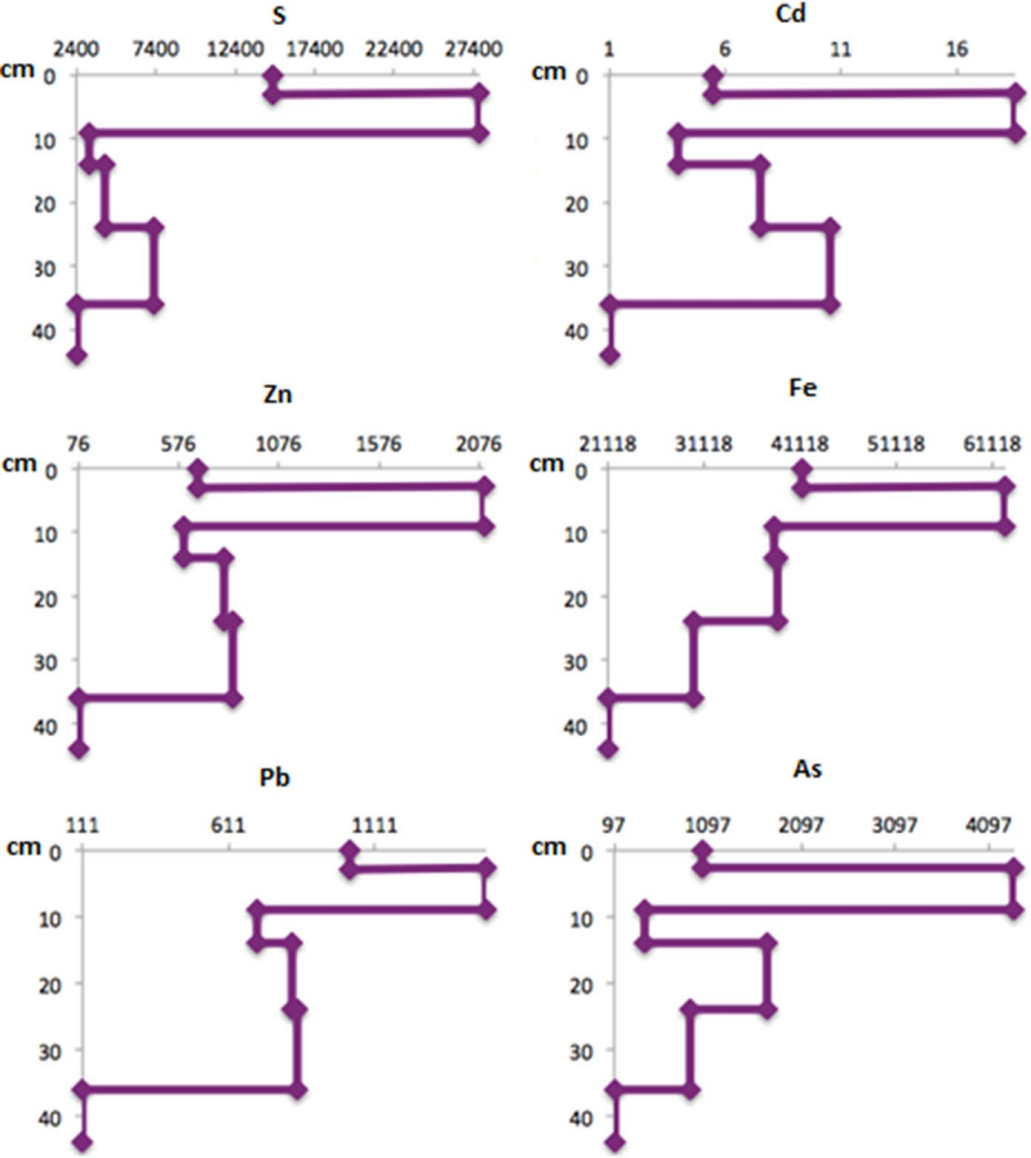
S, Cd, Zn, Fe, Pb, and As concentrations (mg/kg) with depth (cm) in Zim7 soil profile

Similar behavior with depth was observed in the concentrations of S, Cd, Zn, Fe, Pb, and As (Fig. 7) with the highest value in horizon 2 (2.7–9 cm). In addition, the concentrations of As and Pb were above the limits established by Mexican standards NOM-147 and NOM-004 in all the profile horizons except the deepest one (36–44 cm).

### Real and apparent density, porous space, and clay contents

**Fig. 8:**
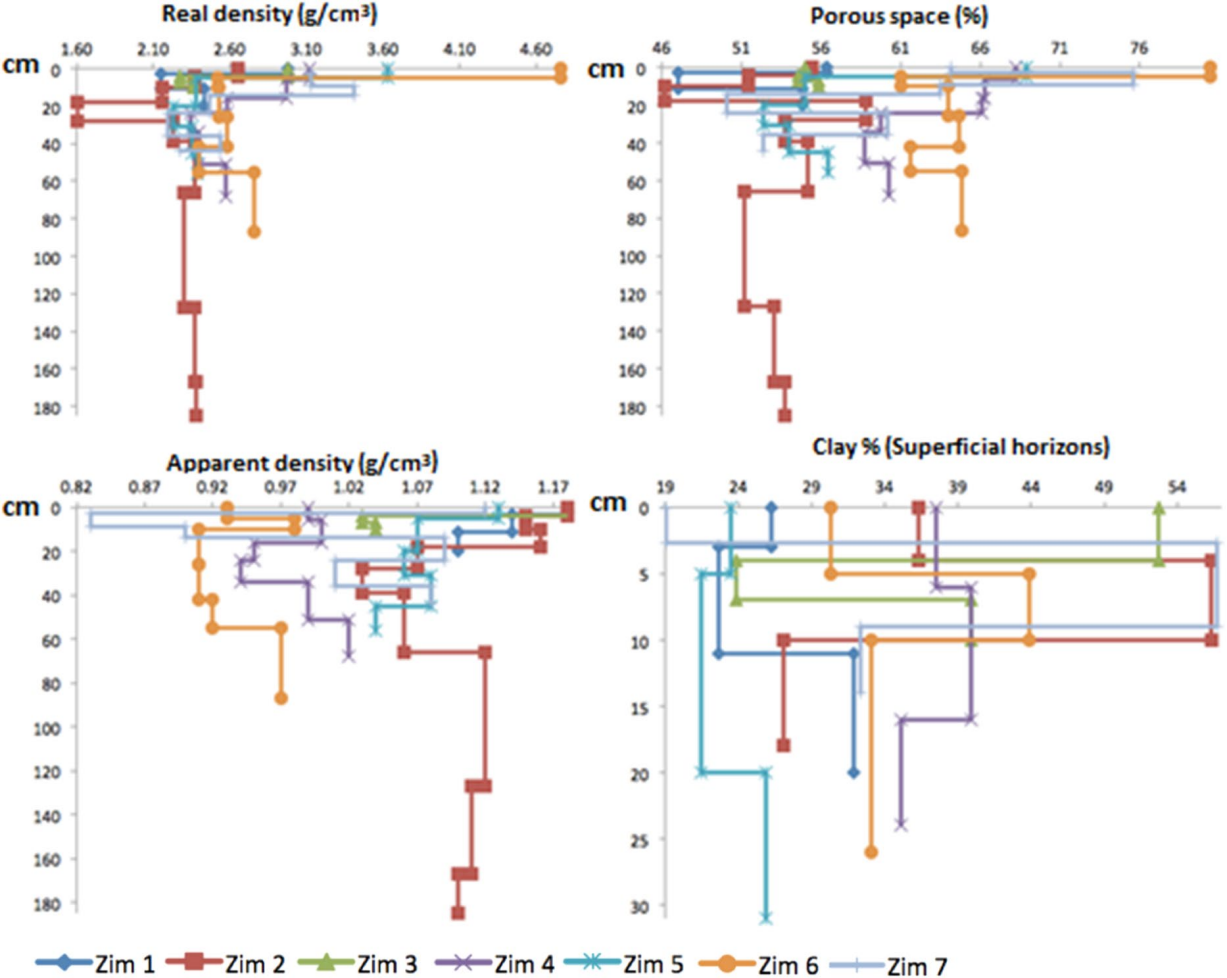
Real density (g/cm^3^), porous space (%), apparent density (g/cm^3^), and clay proportion (%) with depth (cm) in the profiles

The influence of the settling of small particles carried by the wind (which is significant in dry and hot climates as in Zimapán) was expected to affect the physical properties of the soils, like density and porous space. Besides, water was also considered to affect the clay contents in the tailings-impacted soils. Real and apparent density, porous space, and superficial clay contents are shown in Fig. 8. Contrary to expectations (significant clay proportion in tailings), clay percentage does not seem to indicate the possible influence of tailings deposit since the highest contents were measured in samples from Zim 2, Zim 3, and Zim 7 located at different distances without a trend. On the other hand, the highest real density values (up to 4.74 g/cm^3^) were determined in the superficial horizons of the closest sites to the impoundments. Apparent density ranged from 0.82 to 1.17 g/cm^3^, corresponding to relatively low values that may be due to the high calcium carbonate content of the soils. The lowest values were measured in Zim 7, Zim 6, and Zim 4, which also presented the highest porous space values.

### Conductivity and pH

**Fig. 9:**
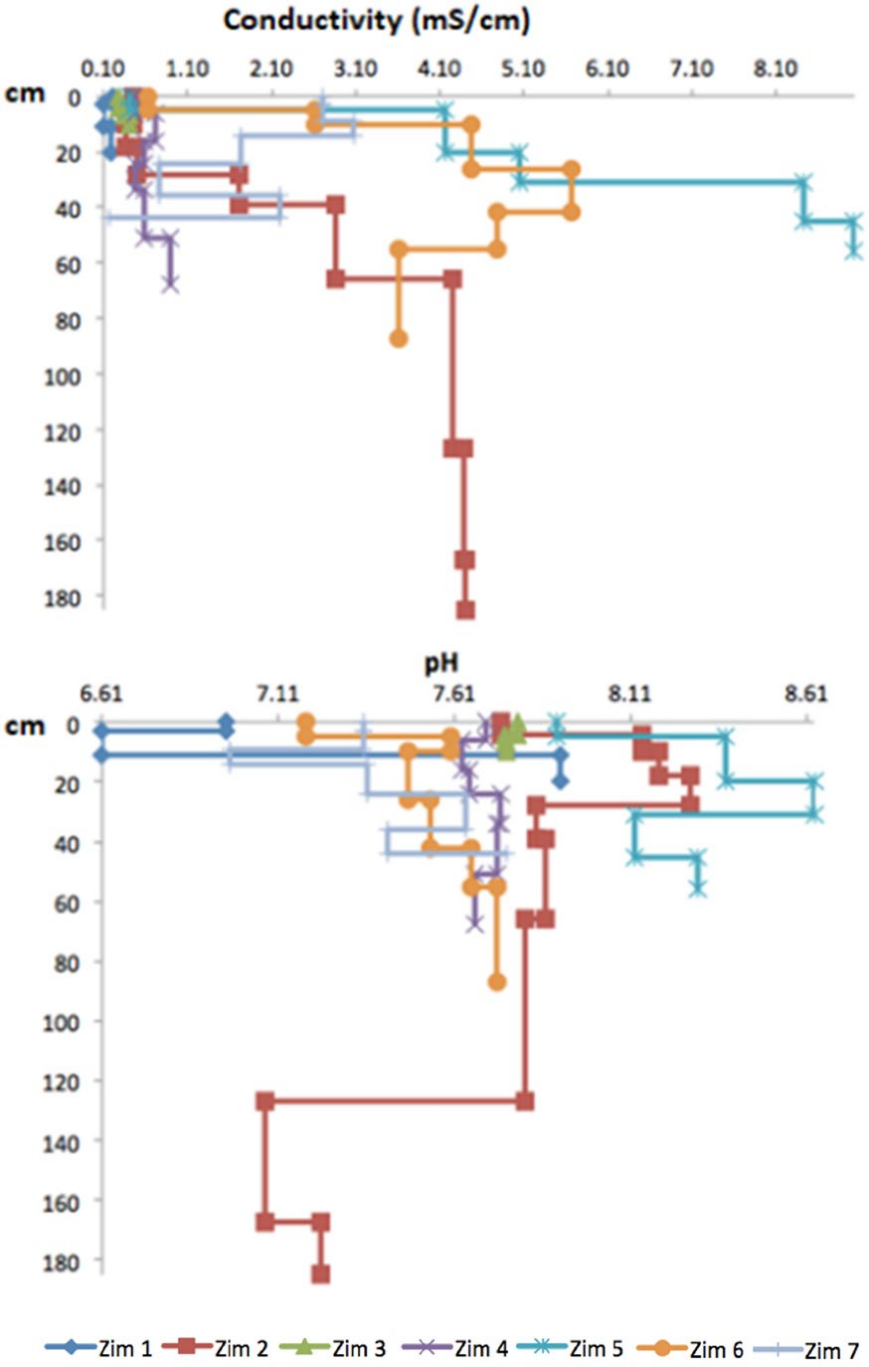
Conductivity (mS/cm) and pH with depth (cm) in the profiles

Conductivity presented contrasting values between the superficial layers of Zim 1, Zim 2, and Zim 3 and those of Zim 5, Zim 6, and Zim 7, but the highest one (9.03 mS/cm) was measured in the deepest horizon of Zim 5 (Fig. 9). A high value (5.67 mS/cm) was also determined in the 4th horizon of Zim 6. Alternatively, pH was mostly alkaline at all the sites without a trend (Fig. 9). The higher superficial conductivity in the closest locations to the impoundments indicates the influence of tailings deposit. In addition, the high conductivity at depth in Zim 6 shows that tailings have affected the soils for a long time.

### Alkalinity and carbonates

**Fig. 10:**
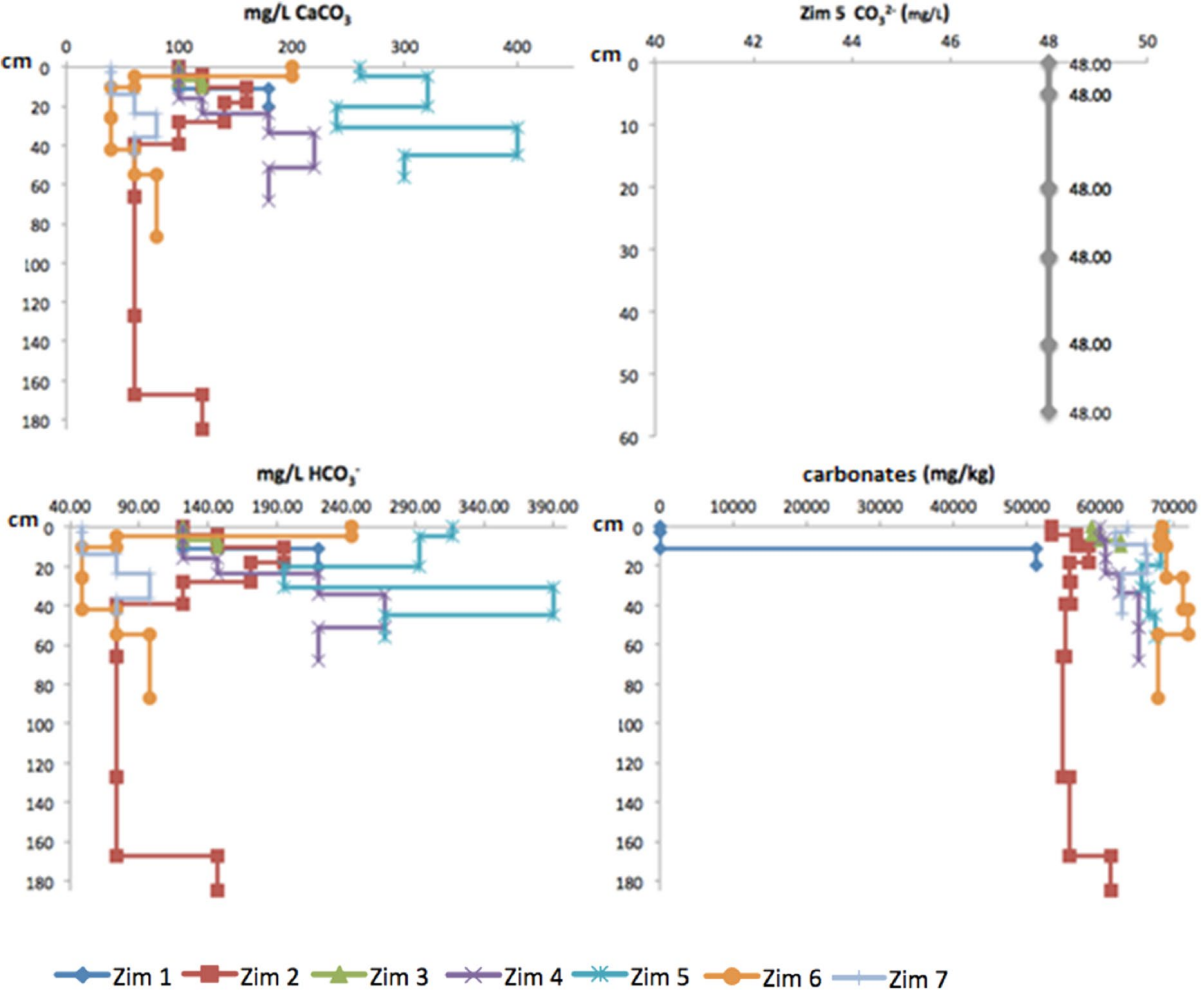
Alkalinity (mg/L CaCO_3_), CO_3_^2−^ (mg/L), HCO_3_^−^ (mg/L), and carbonates (mg/kg) with depth (cm) in the profiles

Alkalinity as calcium carbonate and bicarbonate contents were similar in Zim 1, Zim 2, Zim 3, Zim 4, and Zim 7 profiles with lower values at the surface and a step behavior gradually increasing and decreasing at depth. This similar behavior reflects the abundance of limestones at Zimapán that influence the pH, As, and heavy metals geochemical processes (Espinosa et al., 2009). On the other hand, the highest value in Zim 6 profile was measured at the surface, drastically decreasing at depth. While Zim 5 had a distinct behavior at the surface with more elevated bicarbonate and the presence of carbonates corresponding to its high pH (near 8). Carbonates (directly measured in the solids) were absent in the superficial layer of Zim1 but drastically increased in the last horizon. Low values were also determined in the surface layers of Zim 2, Zim 3, Zim 4, Zim 6, and Zim 7, mainly increasing at depth. Finally, a high carbonate concentration was measured in the first horizon at Zim 5, slightly decreasing with minor variations at depth (Fig. 10).

### Organic matter

**Fig. 11:**
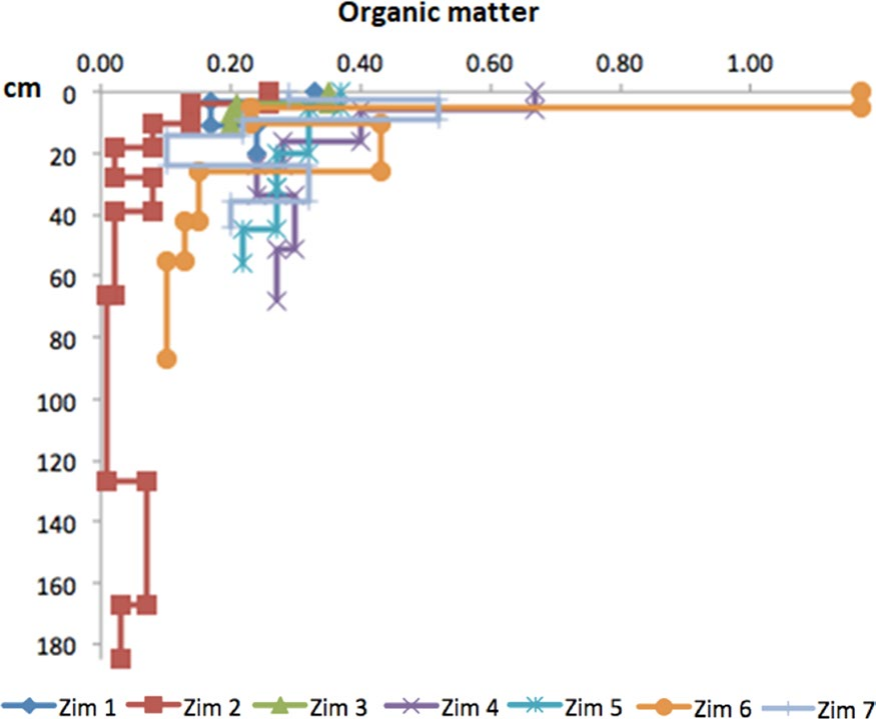
Organic matter (%) with depth (cm) in the profiles

Low values of organic matter were determined in all the profiles (Fig. 11); this may be due to the zones’ climate and type of vegetation with a dominant scrub (INEGI, 2010). The highest content was measured at Zim 6, probably due to its position on the side of a hill, followed by Zim 4 next to a stream bed. The higher contents of organic matter were measured in the superficial layers of the other profiles following the order Zim 5 > Zim 3 > Zim 1 > Zim 2.

### Arsenic and heavy metals

**Fig. 12:**
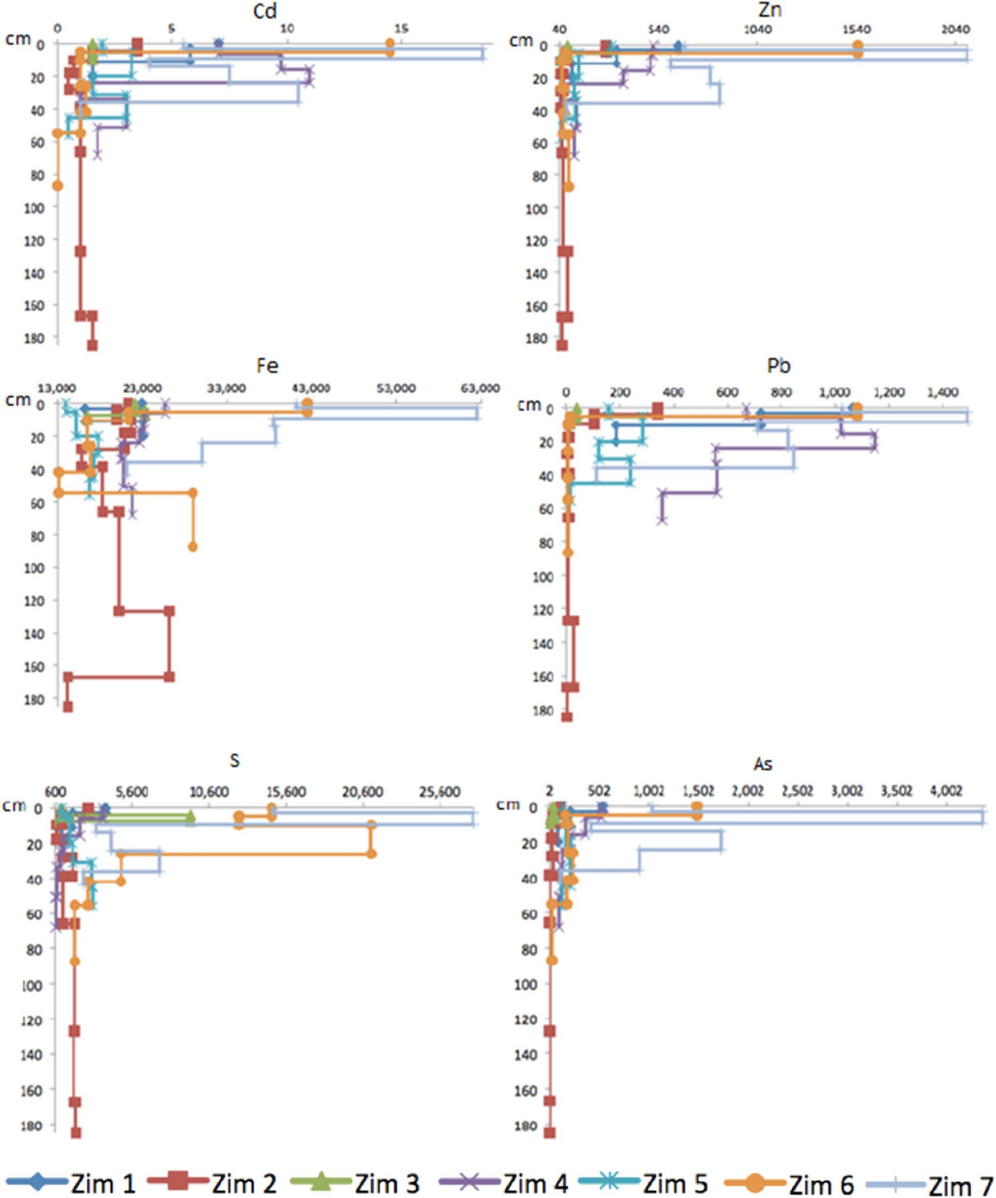
Total concentrations of Cd (mg/kg), Zn (mg/kg), Fe (mg/kg), Pb (mg/kg), S (mg/kg), As (mg/kg) with depth (cm) in the profiles

The highest concentrations of As, S, and the heavy metals Cd, Zn, Fe, Pb were observed in the first horizon of all the profiles (Table 1). Concentrations for each element decreased in the sampled sites as Zim 7 > Zim 6 > Zim 4 > Zim 1 > Zim 5 > Zim 2 > Zim 3 for Cd, Zim 7 > Zim 6 > Zim 1 > Zim 4 > Zim 5 > Zim 2 > Zim 3 for Zn, Zim 7 > Zim 6 > Zim 4 > Zim 1 > Zim 3 > Zim 2 > Zim 5 for Fe, Zim 7 > Zim 4 > Zim 6 > Zim 1 > Zim 5 > Zim 2 > Zim 3 for Pb, Zim 7 > Zim 6 > Zim 4 > Zim 1 > Zim 5 > Zim 2 > Zim 3 for As, and Zim 7 > Zim 6 > Zim 3 > Zim 4 > Zim 1 > Zim 5 > Zim 2 for S (Fig. 12). The highest concentrations of all the PTEs in Zim 7 and Zim 6 were the most remarkable signal of the tailings influence. However, concentrations did not have a decreasing trend with distance since Zim 4 had higher concentrations than Zim 5, probably due to the grabbing of tailing particulates from this site and their accumulation downhill (Fig. 2). The elevated concentration of As and heavy metals at Zim 1 may be due to the tailing particles transported by the wind and settling on the flat zone where Zim 1 was located, in addition to the low rain regime at Zimapán. The lowest PTEs concentrations were measured in Zim 3 located at the Tolimán river shore, probably due to the washing off by the river water.

The concentrations of PTEs in the superficial layer of the profiles are mostly within the range of soils impacted by mining residues at other countries included in Table 2. Particularly, Fe concentrations (up to 42,493 mg/kg) are lower at Zimapán than almost all other mining-impacted sites (up to 450,000 mg/kg in Brazil) reported in Table 2. Zinc concentrations (from 77.5 to 1550 mg/kg) are within the range of most sites except Namibia, Aznalcóllar, and Ciudad Real in Spain. The highest As concentration measured in the surface soil at Zimapán (1484.5 mg/kg) was only surpassed by those reported in Portugal (Everdosa mine), where cassiterite (SnO_2_) is the main ore mineral that was exploited jointly with arsenopyrite (FeAsS) for Sn and As_2_O_3_, respectively (Favas et al., 2011), and also in soils sampled at southern Spain (4293.4 mg/kg) impacted by the tailings and water spill from the Aznalcóllar pyrite mine, occurred on April 25, 1998, that were collected shortly after the event in May 4, 1998 (Simón et al., 2001). Soil cadmium concentrations (from 1.5 to 14.5 mg/kg) determined in our study are comparable to those measured in China (average of 11.0 mg/kg from 72 sites), NE Portugal (7.35 mg/kg), Brazil (1.1 mg/kg), and Cyprus (2.11 mg/kg) (Table 2). Lead contents (from 36 to 1082.5 mg/kg) were lower than those measured in agricultural soils near tailings at the Berg Aukas abandoned mine in Namibia (34,400 mg/kg) and at Ciudad Real, Spain (up to 28,453.5 mg/kg) where soil samples were obtained near the San Quintín mine that was an important Pb–Zn producer during the late nineteenth and early twentieth centuries (Rodríguez et al., 2009).

**Table 2:** Maximum concentrations (mg/kg) of Fe, Zn, As, Cd, Pb in surface layers at each profile (this study) and in soils close to tailings impoundments at other sites

Location	Fe	Zn	As	Cd	Pb	References
Aznalcóllar, Southern Spain	–	8063	4293.4	36.8	9711	Simón et al. (2001)
Cartagena, SE Spain	264,000	2080	1160	82	8310	Bes et al. (2014)
Cyprus	38,700	630	–	2.11	20.35	Christou et al. (2017)
Quadrangle, SE Brazil	450,000	92	43	1.1	28.3	Buch et al. (2021)
Marrakech, Morocco	154,000	391	56.5	0.51	144	El Amari et al. (2014)
Ciudad Real, Spain	–	20,911.8 451.6 (al)	–	54.5 4.89 (al)	28,453.5 970.8 (al)	Rodríguez et al. (2009)
Namibia	–	216,000	–	387	34,400	Kaninga et al. (2020)
Iran	400,000	350.9	35.8	0.33	17.42	Soltani et al. (2017)
Anshan, NE China	–	33	–	0.066	9.5	Zhang et al. (2018)
Aswan, Egypt	–	92	700	0.20	29	Rashed (2010)
China (72 mines)	–	1163*	195.5*	11.0*	641.3*	Li et al. (2014)
NE Portugal	56,600	429	1996	7.35	328	Favas et al. (2011)
Zim 1	22,993	637.5	531.5	7.0	1065	This study
Zim 2	21,368	275	115.8	3.5	340	This study
Zim 3	22,306	77.5	35.9	1.5	36	This study
Zim 4	25,806	512.5	518.9	7.0	670	This study
Zim 5	13,993	300	128.4	2.0	156	This study
Zim 6	42,493	1550	1484.5	14.5	1082.5	This study
Zim 7	41,243	675	1092.5	5.5	1022.5	This study

Regarding Mexican soil regulations, the As concentrations surpassed the agricultural soils limit (22 mg/kg in NOM-147) at all sites. Besides, Pb concentration was above the agricultural soils standard (400 mg/kg) at various depths in Zim 1, Zim 4, and Zim 7, although in some profiles (Zim 2, Zim 6), the limit was only surpassed at the surface. Mexican soil standards do not establish a concentration limit of Fe. At the same time, Zn is only included in NOM-004 (biosolids). Its concentration was below the standards (2800 mg/kg dw classified as excellent and 7500 mg/kg dw classified as good) at all sites. In all samples, cadmium concentration was also below the standard established for agricultural soils of 37 mg/kg (NOM-147). The highest concentrations at the surface of the profiles indicate the important impact of tailings on the soils. Furthermore, the contents of PTEs surpassed other national soil quality standards (Table 3).

**Table 3:** Soil quality standards in various countries

Country	As (mg/kg)	Pb (mg/kg)	Zn (mg/kg)	Cd (mg/kg)	References
Denmark	20^a,b^	40 ^b^	500	0.5^b^	1
Mexico*	22	400		37	2
Australia	20	100	200	2	3
France**	37	400	9000	20	6
Germany**	50	400		20	6
Japan*	150	150		150	3
Norway	2	60	100	3	3
Russia	4.5	55	16	0.76	3
Thailand	30	55	70	0.15	3
Finland	5–100	60–750		1–20	7
UK	32–640	450–750		10–630	8
USA**	22	400	23,000	37	6
Canada*	12	70	250	1.4	4a,b,c,d
Brazil*	35	180	450	3	5
Finland*	5			1	5
China*	30 to 40^+^	50 to 80^+^	200^+^	0.3^+^	5

^a^Based on acute effect, ^b^Based on chronic effect, + pH < 5.6

*Agriculture, **Soil Clean-up Standards for residential land use

(1) Danish Environmental Protection Agency (2002), (2) NOM-147-SEMARNAT/SSA1-2004 (2007), (3) Chen et al., 2018, (4) (a) Canadian Soil Quality Guidelines for the Protection of Environmental and Human Health. Arsenic (Inorganic) 1997, (b) Canadian Soil Quality Guidelines for the Protection of Environmental and Human Health. Lead 1999, Canadian Soil Quality Guidelines for the Protection of Environmental and Human Health. Arsenic (Inorganic) (c) Canadian Soil Quality Guidelines for the Protection of Environmental and Human Health. Zinc 2018, (d) Canadian Soil Quality Guidelines for the Protection of Environmental and Human Health. Cadmium 1999, (5) Buch et al., 2021, (6) Provoost et al., 2006, (7) Ministry of the Environment, Finland (2007), (8) UK Environment Agency (2009)

Comparison of the concentrations measured in the superficial layers of the sampled profiles (except Zim3) shows that As is over most of the limits except those of Japan (150 mg/kg) and UK (from 32 to 640 mg/kg). Pb is above the limits of all countries at most sites, but Zim 5 complies with the values of France, Germany, Finland (highest limit), the UK, the USA, and Brazil, and Zim 2 concentration also complies with the limits of those countries except Brazil. Cadmium surpasses most of the country limits except those of France, Germany, Japan, Finland (highest), the UK (highest), and the USA. Zn is below the limits of France and the USA at all sampled sites but surpasses the limits of Denmark, Australia, and Brazil.

### Mineralogy

The main minerals identified (certainly and probably present) in each horizon are listed in Table 4. Quartz, calcite, anorthite, albite, and muscovite correspond to minerals commonly present in the soils of the area, clays include illite, montmorillonite, nontronite, vermiculite, and saponite. Other minerals correspond to oxides (like hematite), sulfides (like galena), and secondary minerals (like gypsum). Although common soil minerals were present in all the profiles, minerals related to the mineralization at Zimapán (galena, arsenopyrite, sphalerite) and secondary minerals (formed in the tailings) as gypsum and plumboferrite occur mainly in the tailings closest profiles (Zim 5, Zim 6, Zim 7). The presence of arsenopyrite and galena in the deepest horizon of Zim 4 reflects the impact on the soils of decades of tailings accumulation in the area.

**Table 4:** Main minerals identified on the horizons of each soil profile

Horizon	Zim 1	Zim 2	Zim 3	Zim 4	Zim 5	Zim 6	Zim 7
1	Quartz, Anorthite, Hematite, Montmorillonite, Pyromorphite	Quartz, Anorthite, Illite, Calcite, Nontronite	Quartz, Anorthite, Hematite, Montmorillonite, Albite, Orthoclase	Quartz, Hematite, Montmorillonite, Anorthite, Calcite, Illite, Plumboferrite,	Quartz, Anorthite, Calcite, Albite, Sphalerite, Cobaltite, Sulfur	Quartz, Anorthite, Calcite, Cobaltite, Montmorillonite	Quartz, Magnetite, Plumboferrite
2	Quartz, Anorthite, Albite, Gismondine	Quartz, Calcite, Anorthite, Illite, Ankerite	Quartz, Anorthite, Calcite, Galena, Montmorillonite, Muscovite, Periclase	Calcite, Yeelmite, Magnesia, Montmorillonite, Illite, Magnetite	Quartz, Calcite, Albite, Arsenolite, Montmorillonite, Sulfur, Corindon	Anorthite, Calcite, Gypsum, Montmorillonite	Quartz, Bernardite, Gypsum, Montmorillonite
3	Quartz, Albite, Calcite, Arsenolite, Montmorillonite, Muscovite, Dolomite	Quartz, Calcite, Anorthite, Hematite, Magnetite, Ankerite	Quartz, Calcite, Albite, Muscovite, Almandine	Calcite, Anorthite, Albite, Periclase	Quartz, Calcite, Carrollite	Quartz, Anorthite, Calcite, Gypsum, Montmorillonite, Sphalerite, Opal	Quartz, Calcite, Montmorillonite, Sauconite, Orthoclase
4		Quartz, Calcite, Muscovite, Montmorillonite, Illite, Magnetite		Quartz, Anorthite, Calcite, Yeelmite, Magnesium, Corindon	Quartz, Calcite, Cooperite, Sulfur, Montmorillonite, Periclase	Quartz, Calcite, Anorthite	Albite, Calcite, Anorthite, Hematite, Cobaltite, Gypsum, Montmorillonite, Volkonskoite
5		Quartz, Calcite, Montmorillonite, Anorthite, Nontronite, Annite, Muscovite		Calcite, Albite, Montmorillonite, Saponite	Quartz, Calcite, Montmorillonite, Margarite, Truscottite	Quartz, Calcite, Albite	Calcite, Montmorillonite, Vermiculite, Sylvite
6		Quartz, Calcite, Albite, Montmorillonite		Quartz, Calcite, Arsenopyrite, Galena, Montmorillonite, Ankerite		Calcite, Gypsum, Fluorite, Montmorillonite, Vermiculite, Anorthoclase	Quartz, Montmorillonite, Anorthite
7		Quartz, Anorthite, Hematite, Montmorillonite					
8		Quartz, Montmorillonite					
9		Quartz, Albite, Calcite, Montmorillonite, Corindon, Magnetite					

### Statistical correlations and analysis

Pearson correlations among As, heavy metals, S, and the physicochemical parameters (Supplementary material 1) were calculated with SPSS Statistics software to estimate the relations among the measured elements and the parameters. Arsenic showed a positive correlation with the potentially toxic elements (*p* < 0.01, 2-tailed): Pb (*r* = 0.689), Cd (*r* = 0.803), and Zn (*r* = 0.914); however, regarding soil characteristics, it only presented a nearly significant correlation (*r* = 0.493, *p* < 0.01) with porous space. In contrast, Cd and Zn had a positive correlation (*p* < 0.01 2-tailed) with real density (*r* = 0.60 Cd, *r* = 0.663 Zn), porous space (*r* = 0.662 Cd, *r* = 0.621 Zn), and organic matter (*r* = 0.566 Cd, *r* = 0.613 Zn), but a negative one with pH (*r* =  − 0.490 Cd, *r* =  − 0.504 Zn). Lead was also positively correlated (*p* < 0.01, 2-tailed) with Cd (*r* = 0.906), Zn (*r* = 0.824), and porous space (*r* = 0.507) and nearly had a significant correlation with real density (*r* = 0.480), Pb was also negatively correlated (*p* < 0.01, 2-tailed) with pH (*r* =  − 0.525). Iron, conductivity, and sulfur were not significantly correlated with any measured variables.

Regarding soil characteristics, results showed a negative correlation (*p* < 0.01, 2-tailed) between apparent density with porous space (*r* =  − 0.611) and a close to significant (*r* =  − 0.493) with calcium carbonate (mg/kg). Furthermore, real density was correlated (*p* < 0.01, 2-tailed) with organic matter (*r* = 0.793) and porous space with real density (*r* = 0.822) and organic matter (*r* = 0.659). On the other hand, superficial clay content did not significantly correlate with the different soil characteristics.

As expected, calcium carbonate CaCO_3_ (mg/L) and bicarbonate HCO_3_^−^ (mg/L) concentration had a positive correlation (*r* = 0.972, *p* < 0.01, 2-tailed), which was also obtained with pH (*p* < 0.01, 2-tailed) (*r* = 0.549 CaCO_3_, *r* = 0.481HCO_3_^−^). These results indicated the same origin of As and heavy metals except iron, an element common in soils. Besides, some soil characteristics may be related to heavy metals and tailings impact, as discussed below.

The factor analysis (calculated with Statistica software) was performed through the principal component extraction method (Table 5) to corroborate the correlations obtained by employing factors that could explain them. Five representative factors were extracted for the case study (according to the eigenvectors values and the percentage of variance explained). The variables that represent each factor are those with a factor loading > 0.700. According to the analysis, factor 1 represents 41.43% of the variance. The representative variables are RD, PS, As, Pb, Zn, Fe, Cd, S, and OM. The physical characteristics of the soil, such as RD and PS, are directly related to the content of heavy metals and metalloids, as well as S, together with the organic matter content, so this factor represents the direct impact of the mining tailings pollution on the physical characteristics of the soil. Factor 2 explains 16% of the variance of the data, in which the relevant variables are BC (bicarbonates) and CCs (alkalinity) whose correlation with the factor is inverse. Factor 3 represents 12.72% of the variance whose variables describing it are CC and EC. This means that soil’s electrical conductivity is associated with the content of carbonates as CaCO_3_. Factor 4 explains 7.62% of the variance related to the Cl variable (although the value of the factor load does not reach the significance level is very close to it, and it is considered as the variable that describes the factor). The clays do not have a significant relationship with the other variables. The variable that represents the last factor is pH, inversely, with 5.77% of the variance.

**Table 5:** Abstract of the factor analysis of the studied variables (Metals and Metalloids, *CC* Calcium carbonate (mg/kg), *BC* Bicarbonate, *CCs* Alkalinity (mg/L CaCO_3_), *AD* Apparent density, *RD* Real density, *PS* Porous space, *OM* Organic matter, *EC* Conductivity, *Cl* Clay content)

Factor loadings (unrotated) Extraction: principal components (Marked loadings are > .700000)					
	Factor 1	Factor 2	Factor 3	Factor 4	Factor 5
RD	*0.721*	− 0.506	0.187	− 0.254	0.006
AD	− 0.613	− 0.038	0.547	− 0.089	− 0.080
PS	*0.861*	− 0.287	− 0.131	− 0.188	0.056
BC	− 0.310	− *0.907*	0.023	0.004	0.016
Cl	0.282	0.402	− 0.034	− *0.631*	0.346
As	*0.892*	0.119	0.014	0.119	− 0.060
Pb	*0.781*	0.013	0.430	0.275	− 0.029
Zn	*0.948*	− 0.081	0.198	0.079	− 0.063
Fe	*0.887*	0.174	0.040	0.034	− 0.028
Cd	*0.887*	− 0.100	0.300	0.095	0.015
S	*0.759*	0.287	− 0.343	0.106	− 0.145
CC	0.186	− 0.221	− *0.786*	− 0.421	0.025
pH	− 0.116	0.126	0.213	− 0.472	− *0.820*
EC	0.174	0.059	− *0.756*	0.384	− 0.292
OM	*0.701*	− 0.525	0.025	− 0.174	− 0.014
CCs	− 0.329	− *0.865*	− 0.115	0.131	− 0.070
Eigenvalue	6.95	2.56	2.03	1.22	0.92
Cumulative	6.95	9.51	11.54	12.76	13.68
% Total	43.41	16.00	12.72	7.62	5.77
Cumulative	43.41	59.41	72.13	79.75	**85.52**

Variables representing each factor in italics

As can be seen, the content of heavy metals analyzed directly correlates with the physical properties of the soil, which confirms the impact of soil contamination by heavy metals and metalloids from mine tailings on its physical properties.

### Ecological risk

The ecological potential risk indices (RI) were used to evaluate the quality of the soils. This index considers the element contamination factor (Cf), the potential ecological risk factors for each metal (Er), and the sedimentological toxic response factors (Tr) (Hakanson, 1980; Manoj & Padhy, 2014). Although to estimate the Cf, it is necessary to consider the background value of each element of environmental interest. We used those measured in non-polluted sediments collected in the Tolimán River near the tailings zone (Espinosa et al., 2009). The concentrations were: As = 97 mg/kg, Cd = 6 mg/kg, Pb = 76 mg/kg, Zn = 178 mg/kg. Contamination factors and ecological risk factors were calculated by applying the equations:

$${\text{RI }} = \mathop \sum \limits_{i = 1}^{n} {\text{Er}}^{i} = \mathop \sum \limits_{i = 1}^{n} {\text{Tr}}^{i} \times {\text{Cf}}^{i}$$

$${\text{Cf}} = \frac{{{\text{heavy }}\;{\text{metal }}\;{\text{content }}\;{\text{of }}\;{\text{the }}\;{\text{analyzed }}\;{\text{samples}}}}{{{\text{heavy }}\;{\text{metal}}\;{\text{ content}}\;{\text{ from}}\;{\text{ the }}\;{\text{background}}\;{\text{ environment}}}}$$

Tr values were: Cd = 30, As = 10, Fe = 0, Pb = 5, and Zn = 1 (Hakanson, 1980).

The risk grades corresponding to each soil horizon for each profile were estimated according to the values reported in Table 6 (Suresh et al., 2012). Results for each profile and horizon are presented in Supplementary Material 2. Minimum, maximum, and average values for all the profiles are summarized in Table 7 for each element.

**Table 6:** Grading standards of potential and ecological risk index (Suresh et al., 2012)

Er	Risk grade	RI	Risk grade
< 40	Low potential ecological risk	< 150	Low potential ecological risk
40–80	Moderate potential ecological risk	150–300	Moderate potential ecological risk
80–160	Considerable potential ecological risk	300–600	High potential ecological risk
160–320	High potential ecological risk	> 600	Significantly high potential ecological risk
> 320	Significantly high potential ecological risk

**Table 7:** Minimum, maximum, and average values of the contamination factor (Cf), enrichment factor (Er), and ecological risk index (RI) of Cd, As, Fe, Pb, and Zn

	Cfi Cd	Cfi As	Cfi Fe	Cfi Pb	Cfi Zn	sum Cfi	Er Cd	Er As	Er Pb	Er Zn	RI
Min	0.00	0.00	0.59	0.03	0.09	1.26	0.00	0.00	0.16	0.09	3.39
Max	3.08	45.03	2.79	19.61	11.80	82.31	92.50	450.31	98.07	11.80	652.67
Average	0.61	3.93	1.05	4.79	1.68	12.06	18.45	39.33	23.94	1.68	83.39
Stdv	0.71	8.00	0.44	5.67	2.46	16.01	21.34	80.03	28.37	2.46	123.36
Kurtosis	3.44	19.31	6.08	− 0.25	8.23	9.34	3.44	19.31	− 0.25	8.23	11.82
Skewness	1.90	4.07	2.22	0.99	2.70	2.70	1.90	4.07	0.99	2.70	3.05

According to Er values, the calculated factors indicate that As is the only element with a significantly high (450.3 in Zim 7 from 3 to 9 cm) and significantly high potential ecological risk (178.2 in the deepest layer of Zim 7 from 14 to 24 cm). However, Cd and Pb have a considerable potential ecological risk in Zim 7 (92.5 and 98.1, respectively, in the 3–9 cm layer). The surface horizons of Zim1and Zim4 have a moderate potential ecological risk for As (54.8 and 53.5, respectively) and Pb (70.1 and 44.1, respectively), Zim 6 shows a considerable potential ecological risk for As (153.0) and a moderate potential ecological risk for Cd and Pb (72.5 and 71.2, respectively), and the surface layer of Zim 7 has a high potential ecological risk for As (112.6) and moderate potential for Pb (67.3). In contrast, Fe and Zn have a low potential ecological risk in all the profiles and layers. The RI values indicate that only Zim 7 presents a significantly high ecological risk in the 3–9 cm depth layer (652.7), and Zim 6 has a high potential ecological risk (305.5) in the surficial layer. The values also show a moderate potential ecological risk in some of the Zim 1, Zim 4, and Zim 7 layers (Supplementary material 2). Overall, Zim 7 is the profile posing the highest ecological risk.

### General overview

Results evidenced that tailings affect all the sampled sites since the higher As and heavy metals concentrations were measured in the superficial layers indicating the tailings particulates deposit on the soil. In addition, the influence of the parental material in the high PTEs concentrations may be discarded in view of the lower concentrations and observed differences between the lower horizons concerning the shallower ones. In contrast, this influence is reflected in the difference between the real and apparent density mainly due to the presence of high contents of calcium carbonate, which is abundant at Zimapán.

Different tailings dispersion processes impact each site depending on the distance and position in the landscape. For example, settling on Zim 1 and Zim 5 located on the top of the hills, mainly results from wind transport, while Zim 2 and Zim 6 are impacted by wind and rain due to their position on the slopes of the mountains. Besides, Zim 3 is exposed to the wind transport and dragging of the tailings particulates. This dragging may also influence Zim 4, in addition to the influence of the stream water, whose level increases in the rainy season due to heavy storms. On the other hand, Zim 7 is influenced by rainwater (washing from Zim 5 and Zim 6), directly by the wind-carrying particulates from the close-by tailings, and also by acid mine drainage (evidenced by the sulfur peak in the second horizon).

The highest concentrations of arsenic, heavy metals, sulfur, and real density were measured in Zim 6 and Zim 7 profiles closest to the mine wastes, evidencing the tailings influence. The impact of tailings at all sites was also evidenced by the higher contents of those elements in the superficial layers, notwithstanding their distance, soil characteristics, or soil use at each location.

Contrary to expectations (acid pH values due to acid mine drainage), pH had an independent behavior (ranging from 6.9 to 8.6), was not correlated with As (*p* < 0.05), and had a low negative correlation (*p* < 0.01) with Pb (*r* =  − 0.525), Cd(*r* =  − 0.494) and Zn (*r* =  − 0.504). This reflects the geology and soil characteristics of the area, with abundant limestones that rise and neutralize the acid produced by sulfide oxidation. However, the inverse correlation of Zn and Pb with pH reflects the possible deposit and further oxidation of sphalerite and galena that are abundant in tailings (Méndez & Armienta, 2003; Zúñiga-Vázquez et al., 2014) in the superficial horizons.

The presence and abundance of albite, calcite, quartz, anorthite, and montmorillonite, at all sites and most of the horizons indicate these minerals as the primary composition of soils. Secondary minerals, oxides, and sulfides were almost absent in Zim 1, Zim 2, and Zim 3 profiles but increased in the closest (Zim 4, Zim 5, Zim 6, and Zim 7) to tailings profiles where hematite, galena, arsenopyrite, sphalerite, arsenolite, and gypsum were identified. These minerals originally in the ore or resulting from sulfide oxidation indicate tailings dispersion on the soils.

## Conclusions

Real density and porous space showed to be good indicators of tailings dispersion with the highest values in the superficial horizons, notwithstanding the distance and position in the landscape. On the contrary, clay content and pH did not relate to distance from tailings. However, the negative correlation of Zn and Cd with pH shows the occurrence of sulfides oxidation and acid mine drainage generation in superficial layers.

The increase of As, Cd, Pb, Fe, Zn, and S on the superficial horizons of all profiles, mainly in those closest to the impoundments, evidence tailings deposit. Although concentrations generally decreased with distance, a direct trend was not observed. Besides, higher concentrations were measured in the first horizon of the farthest site concerning the second closest to the tailings, showing a different transport mean of tailings particulates.

The soil mineralogy was mainly disturbed by tailings in the closest sites with a higher presence of secondary minerals, oxides, and sulfides. In addition, the presence of sphalerite, arsenolite, and gypsum may be another indicator of the tailings’ impact on soils. General mineralogical characteristics may also be applied as an approximation of the effect of tailings on soils at other sites.

According to ecological risk indicators, As poses the highest ecological risk, followed by Pb and Cd. Arsenic is also an element above Mexican and international soil standards at all the sampled surface horizons.

### Supplementary Information

Below is the link to the electronic supplementary material.

Supplementary file1 (DOCX 29 kb)

Supplementary file2 (DOCX 18 kb)

## Data Availability

The data that support the findings of this study are available on request from the corresponding author.
