# Supplementary material for: Edaphic properties as pieces of evidence of tailings deposit on soils
Source: Environ Geochem Health. 2023 Jun 25;45(12):9175–97. doi: 10.1007/s10653-023-01657-x (PMC10673738; doi:10.1007/s10653-023-01657-x)
Supplement: Supplementary file 1 — Supplementary file1 (DOCX 29 kb) [file 10653_2023_1657_MOESM1_ESM.docx]

SUPPLEMENTARY MATERIAL 1

TABLE S1. Pearson correlations among the analyzed elements and soil characteristics. Bilateral significance. Elements correspond to their symbol. CC= Calcium carbonate (mg/kg), BC =Bicarbonate, CCs = Alkalinity (mg/L CaCO_3_), AD = Apparent density, RD = Real density, PS= Porous space, OM = Organic matter,

mS/cm= Conductivity, Cl=Clay content. N= Number of data

|  |  | As | Ca | CC | BC | Cd | CCs | AD | RD | PS | Fe | OM | mS/cm | Pb | pH | Zn | S | Cl |
| --- | --- | --- | --- | --- | --- | --- | --- | --- | --- | --- | --- | --- | --- | --- | --- | --- | --- | --- |
|  | Corr | 1 | .* | -0.168 | -0.188 | 0.803** | 0.147 | -0.347** | 0.454** | 0.493** | 0.055 | 0.430** | 0.017 | 0.689 | -0.393* | 0.914** | 0.248 | 0.336 |
| As | Sig. (2-tailed) |  | . | 0.315 | 0.257 | 0 | 0.392 | 0.033 | 0.004 | 0.002 | 0.743 | 0.007 | 0.920 | 0.000 | 0.015 | 0.000 | 0.134 | 0.137 |
|  | N | 38 | 5 | 38 | 38 | 37 | 36 | 38 | 38 | 38 | 38 | 38 | 38 | 38 | 38 | 38 | 38 | 21 |
|  | Corr | .* | .* | .* | .* | .* | .* | .* | .* | .* | .* | .* | .* | .* | .* | .* | .* | .* |
| Ca | Sig. (2-tailed) | . |  | . | . |  | . | . |  | . | . |  | . | . |  | . | . |  |
|  | N | 5 | 5 | 5 | 5 | 5 | 5 | 5 | 5 | 5 | 5 | 5 | 5 | 5 | 5 | 5 | 5 | 3 |
|  | Corr | -0.168 | .* | 1 | 0.972** | -0.135 | 0.191 | 0.135 | 0.025 | -0.094 | -0.062 | 0.182 | 0.291 | -0.117 | 0.549** | -0.174 | -0.147 | -0.394 |
| CC | Sig. (2-tailed) | 0.315 | . |  | 0.000 | 0.427 | 0.265 | 0.418 | 0.883 | 0.576 | 0.714 | 0.274 | 0.076 | 0.485 | 0.000 | 0.297 | 0.379 | 0.077 |
|  | N | 38 | 5 | 38 | 38 | 37 | 36 | 38 | 38 | 38 | 38 | 38 | 38 | 38 | 38 | 38 | 38 | 21 |
|  | Corr | -0.188 | .* | 0.972** | 1 | -0.116 | 0.132 | 0.152 | 0.1 | -0.044 | -0.054 | 0.201 | 0.124 | -0.087 | 0.481** | -0.161 | -0.155 | -0.360 |
| BC | Sig. (2-tailed) | 0.257 | . | 0 |  | 0.493 | 0.444 | 0.362 | 0.55 | 0.793 | 0.747 | 0.226 | 0.459 | 0.601 | 0.002 | 0.336 | 0.354 | 0.109 |
|  | N | 38 | 5 | 38 | 38 | 37 | 36 | 38 | 38 | 38 | 38 | 38 | 38 | 38 | 38 | 38 | 38 | 21 |
|  | Corr | 0.803** | .* | -0.135 | -0.116 | 1 | 0.102 | -0.315 | 0.601** | 0.622** | 0.147 | 0.566** | -0.195 | 0.906** | -0.494** | 0.925** | 0.309 | 0.215 |
| Cd | Sig. (2-tailed) | 0 | . | 0.427 | 0.493 |  | 0.560 | 0.057 | 0 | 0 | 0.385 | 0.000 | 0.248 | 0.000 | 0.002 | 0.000 | 0.062 | 0.349 |
|  | N | 37 | 5 | 37 | 37 | 37 | 35 | 37 | 37 | 37 | 37 | 37 | 37 | 37 | 37 | 37 | 37 | 21 |
|  | Corr | 0.147 | .* | 0.191 | 0.132 | 0.102 | 1 | -0.493** | 0.224 | 0.408* | 0.009 | 0.234 | 0.402* | 0.091 | -0.077 | 0.129 | 0.075 | -0.308 |
| CCs | Sig. (2-tailed) | 0.392 | . | 0.265 | 0.444 | 0.560 |  | 0.002 | 0.189 | 0.013 | 0.957 | 0.169 | 0.015 | 0.597 | 0.654 | 0.452 | 0.663 | 0.199 |
|  | N | 36 | 5 | 36 | 36 | 35 | 36 | 36 | 36 | 36 | 36 | 36 | 36 | 36 | 36 | 36 | 36 | 19 |
|  | Corr | -0.347* | .* | 0.135 | 0.152 | -0.315 | -0.493** | 1 | -0.138 | -0.611** | 0.389* | -0.200 | 0.232 | -0.210 | 0.113 | -0.299 | -0.025 | -0.120 |
| AD | Sig. (2-tailed) | 0.033 | . | 0.418 | 0.362 | 0.057 | 0.002 |  | 0.410 | 0 | 0.016 | 0.229 | 0.160 | 0.206 | 0.501 | 0.068 | 0.891 | 0.604 |
|  | N | 38 | 5 | 38 | 38 | 37 | 36 | 38 | 38 | 38 | 38 | 38 | 38 | 38 | 38 | 38 | 38 | 21 |
|  | Corr | 0.454** | .* | 0.025 | 0.1 | 0.601** | 0.224 | -0.138 | 1 | 0.822** | 0.135 | 0.793** | -0.189 | 0.480** | 0.309 | 0.663** | -0.091 | 0.092 |
| RD | Sig. (2-tailed) | 0.004 | . | 0.883 | 0.55 | 0 | 0.189 | 0.410 |  | 0 | 0.418 | 0.000 | 0.256 | 0.002 | 0.059 | 0.000 | 0.586 | 0.693 |
|  | N | 38 | 5 | 38 | 38 | 37 | 36 | 38 | 38 | 38 | 38 | 38 | 38 | 38 | 38 | 38 | 38 | 21 |
|  | Corr | 0.493** | .* | -0.094 | -0.044 | 0.622** | 0.408* | -0.611** | 0.822** | 1 | -0.039 | 0.659** | -0.026 | 0.507** | -0.292 | 0.621** | -0.036 | 0.171 |
| PS | Sig. (2-tailed) | 0.002 | . | 0.576 | 0.793 | 0 | 0.013 | 0 | 0 |  | 0.814 | 0.000 | 0.876 | 0.001 | 0.075 | 0.000 | 0.695 | 0.459 |
|  | N | 38 | 5 | 38 | 38 | 37 | 36 | 38 | 38 | 38 | 38 | 38 | 38 | 38 | 38 | 38 | 38 | 21 |
|  | Corr | 0.055 | .* | -0.062 | -0.054 | 0.147 | 0.009 | 0.389* | 0.135 | -0.039 | 1 | 0.090 | 0.147 | 0.289 | -0.304 | 0.152 | -0.031 | -0.155 |
| Fe | Sig. (2-tailed) | 0.743 | . | 0.714 | 0.747 | 0.385 | 0.957 | 0.016 | 0.418 | 0.814 |  | 0.591 | 0.378 | 0.078 | 0.064 | 0.361 | 0.83 | 0.504 |
|  | N | 38 | 5 | 38 | 38 | 37 | 36 | 38 | 38 | 38 | 38 | 38 | 38 | 38 | 38 | 38 | 38 | 21 |
|  | Corr | 0.430** | .* | 0.182 | 0.201 | 0.566** | 0.234 | -0.200 | 0.793** | 0.659** | 0.090 | 1 | -0.066 | 0.461** | -0.233 | 0.613** | -0.017 | -0.007 |
| OM | Sig. (2-tailed) | 0.007 | . | 0.274 | 0.226 | 0.000 | 0.169 | 0.229 | 0.000 | 0.000 | 0.591 | 38 | 0.692 | 0.004 | 0.179 | 0.000 | 0.919 | 0.977 |
|  | N | 38 | 5 | 38 | 38 | 37 | 36 | 38 | 38 | 38 | 38 |  | 38 | 38 | 38 | 38 | 38 | 21 |
|  | Corr | 0.017 | .* | 0.291 | 0.124 | -0.195 | 0.402* | 0.232 | -0.189 | -0.026 | 0.147 | -0.066 | 1 | -0.304 | 0.21 | -0.152 | -0.053 | -0.106 |
| ms/cm | Sig. (2-tailed) | 0.920 | . | 0.076 | 0.459 | 0.248 | 0.015 | 0.160 | 0.256 | 0.876 | 0.378 | 0.692 |  | 0.063 | 0.206 | 0.363 | 0.751 | 0.648 |
|  | N | 38 | 5 | 38 | 38 | 37 | 36 | 38 | 38 | 38 | 38 | 38 | 38 | 38 | 38 | 38 | 38 | 21 |
|  | Corr | 0.689** | .* | -0.117 | -0.087 | 0.906** | 0.091 | -0.210 | 0.480** | 0.507** | 0.289 | 0.461** | -0.304 | 1 | -0.525** | 0.824** | 0.256 | 0.025 |
| Pb | Sig. (2-tailed) | 0 | . | 0.485 | 0.601 | 0.000 | 0.597 | 0.206 | 0.002 | 0.001 | 0.078 | 0.004 | 0.063 |  | 0.001 | 0.000 | 0.122 | 0.915 |
|  | N | 38 | 5 | 38 | 38 | 37 | 36 | 38 | 38 | 38 | 38 | 38 | 38 | 38 | 38 | 38 | 38 | 21 |
|  | Corr | -0393* | .* | 0.549** | 0.481** | -0.494** | -0.077 | 0.113 | 0.309 | -0.292 | -0.304 | -0.233 | 0.21 | -0.525** | 1 | -0.504** | -0.106 | -0.024 |
| pH | Sig. (2-tailed) | 0.015 | . | 0.00 | 0.002 | 0.002 | 0.654 | 0.501 | 0.059 | 0.075 | 0.064 | 0.179 | 0.206 | 0.001 |  | 0.001 | 0.528 | 0.918 |
|  | N | 38 | 5 | 38 | 38 | 37 | 36 | 38 | 38 | 38 | 38 | 38 | 38 | 38 | 38 | 38 | 38 | 21 |
|  | Corr | 0.914** | .* | -0.174 | -0.161 | 0.925** | 0.129 | -0.299 | 0.663** | 0.621** | 0.152 | 0.613** | -0.152 | 0.824** | -0.504** | 1 | 0.283 | 0.209 |
| Zn | Sig. (2-tailed) | 0 | . | 0.297 | 0.336 | 0.000 | 0.452 | 0.068 | 0.000 | 0.000 | 0.361 | 0.000 | 0.363 | 0.000 | 0.001 |  | 0.085 | 0.364 |
|  | N | 38 | 5 | 38 | 38 | 37 | 36 | 38 | 38 | 38 | 38 | 38 | 38 | 38 | 38 | 38 | 38 | 21 |
|  | Corr | 0.248 | .* | -0.147 | -0.155 | 0.309 | 0.075 | -0.025 | -0.091 | -0.036 | -0.031 | -0.017 | -0.053 | 0.256 | -0.106 | 0.283 | 1 | 0.192 |
| S | Sig. (2-tailed) | 0.134 | . | 0.379 | 0.354 | 0.062 | 0.663 | 0.891 | 0.586 | 0.695 | 0.83 | 0.919 | 0.751 | 0.122 | 0.528 | 0.085 |  | 0.404 |
|  | N | 38 | 5 | 38 | 38 | 37 | 36 | 38 | 38 | 38 | 38 | 38 | 38 | 38 | 38 | 38 | 38 | 21 |
|  | Corr | 0.336 | .* | -0.394 | -0.360 | 0.215 | -0.308 | -0.120 | 0.092 | 0.171 | -0.155 | -0.007 | -0.106 | 0.025 | -0.024 | 0.209 | 0.192 | 1 |
| Cl | Sig. (2-tailed) | 0.137 | . | 0.077 | 0.109 | 0.349 | 0.199 | 0.604 | 0.693 | 0.459 | 0.504 | 0.977 | 0.648 | 0.915 | 0.918 | 0.364 | 0.404 |  |
|  | N | 21 | 3 | 21 | 21 | 21 | 19 | 21 | 21 | 21 | 21 | 21 | 21 | 21 | 21 | 21 | 21 | 21 |
