# Supplementary material for: Edaphic properties as pieces of evidence of tailings deposit on soils
Source: Environ Geochem Health. 2023 Jun 25;45(12):9175–97. doi: 10.1007/s10653-023-01657-x (PMC10673738; doi:10.1007/s10653-023-01657-x)
Supplement: Supplementary file 2 — Supplementary file2 (DOCX 18 kb) [file 10653_2023_1657_MOESM2_ESM.docx]

Supplementary Material 2

Table S2. Contamination factor (Cf), Enrichment factor (Er), and Ecological Risk Index (RI) of Cd, As, Fe, Pb, and Zn for each profile and layer. Green = low potential ecological risk, yellow= moderate potential ecological risk, brown= considerable potential ecological risk, leather= high potential ecological risk, red= significantly high potential ecological risk

|  | Cfi Cd | Cfi As | Cfi Fe | Cfi Pb | Cfi Zn | Σ Cfi | Er Cd | Er As | Er Fe | Er Pb | Er Zn | RI |
| --- | --- | --- | --- | --- | --- | --- | --- | --- | --- | --- | --- | --- |
| **Zim1**(Ap) 0-3 cm | 1.2 | 5.5 | 1.0 | 14.0 | 3.6 | 25.27 | 35 | 54.8 | 0.0 | 70.1 | 3.6 | 163.4 |
| Zim1 (A) 3-11 cm | 1.0 | 2.1 | 0.7 | 9.5 | 1.8 | 15.17 | 29 | 21.2 | 0.0 | 47.7 | 1.8 | 99.7 |
| Zim1 (AB) 11-20 cm | 0.3 | 0.8 | 1.0 | 2.4 | 0.6 | 5.05 | 7.5 | 7.8 | 0.0 | 12.2 | 0.6 | 28.0 |
| **Zim2** (Ah) 0-4 cm | 0.6 | 1.2 | 1.0 | 4.5 | 1.5 | 8.75 | 17.5 | 11.9 | 0.0 | 22.4 | 1.5 | 53.4 |
| Zim2 (A) 4-10 cm | 0.3 | 0.3 | 0.9 | 1.4 | 0.4 | 3.25 | 7.5 | 3.2 | 0.0 | 6.8 | 0.4 | 17.9 |
| Zim2 (AB) 10-18 cm | 0.1 | 0.3 | 1.0 | 0.1 | 0.3 | 1.78 | 4 | 2.5 | 0.0 | 0.7 | 0.3 | 7.6 |
| Zim2 (B1) 18-28 cm | 0.1 | 0.2 | 0.9 | 0.1 | 0.3 | 1.68 | 2.5 | 2.3 | 0.0 | 0.5 | 0.3 | 5.6 |
| Zim2 (B2) 28-39 cm | 0.2 | 0.3 | 0.7 | 0.0 | 0.3 | 1.46 | 5 | 2.9 | 0.0 | 0.2 | 0.3 | 8.3 |
| Zim2 (BC1) 39-66cm | 0.2 | 0.0 | 0.8 | 0.1 | 0.3 | 1.44 | 5 | 0.4 | 0.0 | 0.6 | 0.3 | 6.3 |
| Zim2 (BC2) 66-127 cm | 0.2 | 0.0 | 0.9 | 0.1 | 0.3 | 1.51 | 5 | 0.3 | 0.0 | 0.5 | 0.3 | 6.1 |
| Zim2 (BCk) 127-167 cm | 0.2 | 0.1 | 1.2 | 0.3 | 0.4 | 2.14 | 5 | 0.5 | 0.0 | 1.6 | 0.4 | 7.5 |
| Zim2 (C) 167-185 cm | 0.3 | 0.0 | 0.6 | 0.0 | 0.3 | 1.26 | 7.5 | 0.3 | 0.0 | 0.2 | 0.3 | 8.3 |
| **Zim3** (A) 0-4 cm | 0.3 | 0.4 | 1.0 | 0.5 | 0.4 | 2.52 | 7.5 | 3.7 | 0.0 | 2.4 | 0.4 | 14.0 |
| Zim3 (B) 4-7 cm | 0.3 | 0.4 | 1.0 | 0.5 | 0.5 | 2.69 | 7.5 | 4.3 | 0.0 | 2.5 | 0.5 | 14.8 |
| Zim3 (BC) 7-40 cm | 0.3 | 0.1 | 0.7 | 0.2 | 0.3 | 1.52 | 7.5 | 0.8 | 0.0 | 1.0 | 0.3 | 9.5 |
| **Zim4** (Ap) 0-6 cm | 1.2 | 5.3 | 1.2 | 8.8 | 2.9 | 19.36 | 35 | 53.5 | 0.0 | 44.1 | 2.9 | 135.5 |
| Zim4 (A) 6-16 cm | 1.6 | 3.7 | 1.0 | 13.4 | 2.8 | 22.55 | 49 | 36.8 | 0.0 | 66.9 | 2.8 | 155.6 |
| Zim4 (B1) 16-24 cm | 1.9 | 2.1 | 1.0 | 15.1 | 2.0 | 22.15 | 55.5 | 21.3 | 0.0 | 75.6 | 2.0 | 154.4 |
| Zim4 (B2) 24-34 cm | 0.2 | 1.2 | 0.9 | 7.3 | 0.1 | 9.70 | 5 | 12.3 | 0.0 | 36.5 | 0.1 | 53.9 |
| Zim4 (BC) 34-51 cm | 0.5 | 1.2 | 0.9 | 7.4 | 0.7 | 10.72 | 15 | 11.9 | 0.0 | 37.0 | 0.7 | 64.6 |
| Zim4 (C) 51-68 cm | 0.3 | 0.0 | 1.0 | 4.7 | 0.6 | 6.61 | 9 | 0.0 | 0.0 | 23.5 | 0.6 | 33.2 |
| **Zim5** (Ap) 0-5 cm | 0.3 | 1.3 | 0.6 | 2.1 | 1.7 | 6.02 | 10 | 13.2 | 0.0 | 10.3 | 1.7 | 35.2 |
| Zim5 (BA) 5-20 cm | 0.5 | 1.7 | 0.7 | 3.7 | 0.8 | 7.43 | 16 | 17.3 | 0.0 | 18.6 | 0.8 | 52.7 |
| Zim5 (Bc) 20-31 cm | 0.3 | 2.2 | 0.8 | 1.6 | 0.6 | 5.50 | 7.5 | 22.3 | 0.0 | 8.0 | 0.6 | 38.4 |
| Zim5 (BC) 31-45 cm | 0.5 | 5.5 | 0.8 | 3.1 | 0.6 | 10.56 | 15 | 55.1 | 0.0 | 15.7 | 0.6 | 86.4 |
| Zim5 (CB) 45-56 cm | 0.1 | 1.2 | 0.8 | 0.2 | 0.3 | 2.58 | 2.5 | 12.1 | 0.0 | 1.0 | 0.3 | 16.0 |
| **Zim6** (Ah) 0-5 cm | 2.4 | 15.3 | 1.9 | 14.2 | 8.7 | 42.57 | 72.5 | 153.0 | 0.0 | 71.2 | 8.7 | 305.5 |
| Zim6 (BA) 5-10 cm | 0.2 | 1.6 | 1.0 | 0.2 | 0.4 | 3.28 | 5 | 15.8 | 0.0 | 1.0 | 0.4 | 22.2 |
| Zim6 (B1) 10-26 cm | 0.2 | 2.0 | 0.7 | 0.1 | 0.3 | 3.26 | 5 | 19.9 | 0.0 | 0.3 | 0.3 | 25.5 |
| Zim6 (B2) 26-42 cm | 0.2 | 2.4 | 0.8 | 0.1 | 0.4 | 3.75 | 6 | 23.6 | 0.0 | 0.3 | 0.4 | 30.3 |
| Zim6 (BC) 42-55 cm | 0.2 | 1.8 | 0.6 | 0.1 | 0.3 | 2.98 | 5 | 18.3 | 0.0 | 0.4 | 0.3 | 24.0 |
| Zim6 (C) 55-87 cm | 0.0 | 0.2 | 1.3 | 0.1 | 0.5 | 2.10 | 0 | 2.4 | 0.0 | 0.5 | 0.5 | 3.4 |
| **Zim7** (Ag) 0-3 cm | 0.9 | 11.3 | 1.8 | 13.5 | 3.8 | 31.27 | 27.5 | 112.6 | 0.0 | 67.3 | 3.8 | 211.2 |
| Zim7 (Cg) 3-9 cm | 3.1 | 45.0 | 2.8 | 19.6 | 11.8 | 82.31 | 92.5 | 450.3 | 0.0 | 98.1 | 11.8 | 652.7 |
| Zim7 (CBg) 9-14 cm | 0.7 | 4.4 | 1.7 | 9.3 | 3.4 | 19.49 | 20 | 44.0 | 0.0 | 46.7 | 3.4 | 114.0 |
| Zim7 (BCg1) 14-24 cm | 1.3 | 17.8 | 1.7 | 10.9 | 4.5 | 36.15 | 37.5 | 178.2 | 0.0 | 54.3 | 4.5 | 274.5 |
| Zim7 (BCg2) 24-36 cm | 1.8 | 9.3 | 1.3 | 11.2 | 4.8 | 28.36 | 52.5 | 93.4 | 0.0 | 55.8 | 4.8 | 206.4 |
| Zim7 (CB) 36-44 cm | 0.2 | 1.0 | 0.9 | 1.5 | 0.4 | 4.01 | 5 | 10.0 | 0.0 | 7.4 | 0.4 | 22.8 |
